# Development of miRNA-Based Approaches to Explore the Interruption of Mosquito-Borne Disease Transmission

**DOI:** 10.3389/fcimb.2021.665444

**Published:** 2021-06-21

**Authors:** Tie-Long Xu, Ya-Wen Sun, Xin-Yu Feng, Xiao-Nong Zhou, Bin Zheng

**Affiliations:** ^1^ Evidence-Based Medicine Research Center, Jiangxi University of Chinese Medicine, Nanchang, China; ^2^ National Institute of Parasitic Diseases, Chinese Center for Disease Control and Prevention, and WHO Collaborating Center for Tropical Diseases, Shanghai, China; ^3^ Key Laboratory of Parasite and Vector Biology, Ministry of Public Health, Shanghai, China; ^4^ School of Global Health, Chinese Center for Tropical Diseases Research, Shanghai Jiao Tong University School of Medicine, Shanghai, China

**Keywords:** miRNAs, mosquito, *Plasmodium*, interruption, miRNA-based approach, mosquito-borne diseases

## Abstract

MicroRNA (miRNA or miR)-based approaches to interrupt the transmission of mosquito-borne diseases have been explored since 2005. A review of these studies and areas in which to proceed is needed. In this review, significant progress is reviewed at the level of individual miRNAs, and miRNA diversification and relevant confounders are described in detail. Current miRNA studies in mosquitoes include four steps, namely, identifying miRNAs, validating miRNA-pathogen interactions, exploring action mechanisms, and performing preapplication investigations. Notably, regarding the *Plasmodium* parasite, mosquito miRNAs generally bind to mosquito immunity- or development-related mRNAs, indirectly regulating *Plasmodium* infection; However, regarding arboviruses, mosquito miRNAs can bind to the viral genome, directly modifying viral replication. Thus, during explorations of miRNA-based approaches, researchers need select an ideal miRNA for investigation based on the mosquito species, tissue, and mosquito-borne pathogen of interest. Additionally, strategies for miRNA-based approaches differ for arboviruses and protozoan parasites.

## Background

Insecticide-based interventions [e.g., long-lasting insecticide-treated bed nets (LLINs) and indoor residual spraying (IRS)] are important components of integrated mosquito management programs designed to block the transmission of mosquito-borne diseases. The insecticides used in these interventions exert strong selection pressure on resistance and lead to the evolution and spread of mosquito resistance, representing a major concern in mosquito-borne disease control programs (Xu et al., 2014). The World Health Organization (WHO) claims that innovative vector control tools are urgently needed (World Health Organization, 2012). MicroRNAs (miRNAs) are single-stranded, conserved, and small endogenous noncoding RNAs that have important regulatory functions at the posttranscriptional level in diverse organisms (Bartel, 2004; Bartel, 2009). The regulation of miRNAs is indispensable for various processes, including apoptosis, development, differentiation, viral infection, and so on (Bartel, 2004; Bartel, 2009). More importantly, the functions of miRNAs can be explored and utilized. For instance, miR-15, miR-16, miR-34, and Let-7 have been patented and approved for cancer diagnosis or treatment (Mishra et al., 2016). Given their characteristics and functions, miRNAs represent one possibility for establishing a new tool, namely, miRNA-based approaches. Thus, numerous studies on mosquito miRNAs have been performed since 2005 (Wang et al., 2005) with the ultimate goal of utilizing miRNA-based approaches for disease control (Liu et al., 2017; Feng et al., 2018a). The development of a mosquito miRNA-based approach is presumed to always follow the research roadmap of identifying mosquito miRNAs, observing miRNA-pathogen interaction, exploring action mechanisms, performing preapplication investigations and conducting clinical or field trials. Advances at each step of the roadmap need to be outlined to provide important insight into potential applications.

## The Mosquito miRNA Databases Analyzed

Publications focused on investigating mosquito global miRNA profiles were selected for extraction of information, including the authors, publication year, study materials, methods, miRNA names, canonical sequences, and so on. Then, this information was supplemented during the review of the other included papers. Approximately 1635 mature or predicted miRNAs were collected. Of the 1635 miRNAs, 853 (52.17%) were limited to identification and lacked any additional study information. The remaining 782 (47.83%) were further investigated; thus, they were tracked by their annotated names for study development.

Overall, the miRNAs of 24 mosquito species, including 2 species of *Aedes* (Li et al., 2009; Akbari et al., 2013; Gu et al., 2013; Campbell et al., 2014; Hu et al., 2015; Maharaj et al., 2015; Liu et al., 2015; Liu Y. X. et al., 2016; Batz et al., 2017; Su et al., 2017; Zhang et al., 2017), 2 species of *Culex* (Skalsky et al., 2010; Hong et al., 2014), and 20 species of *Anopheles* mosquitoes (Wang et al., 2005; Chatterjee and Chaudhuri, 2006; Mead and Tu, 2008; Dritsou et al., 2014; Liu et al., 2014; Allam et al., 2016; Liu et al., 2017; Carissimo et al., 2018; Feng et al., 2018a; Bruno et al., 2019), were studied. The study materials involved almost every possible type, including mosquito genome sequences obtained from websites; whole mosquitoes at all developmental stages; mosquitoes at different blood feeding states, ages, pathogen infection statuses, diapause statuses or insecticide resistance states; mosquito tissues except for legs; and even mosquito cell nuclei and cytoplasm (**Tables 1** **–** **6**).

**Table 1:** The generally most highly expressed miRNAs in mosquitoes.

miRNA names	References and study materials
miR-1-3p	*Ae. aegypti* (Yen et al., 2018), adult carcasses of *Ae. aegypti* (Akbari et al., 2013)
miR-10-5p	*An. gambiae* (Biryukova et al., 2014), *An. sinensis* (Feng et al., 2018a)
miR-124-3p	*Ae. aegypti*, *Cx. quinquefasciatus* and *An. gambiae* (Behura et al., 2011)
miR-125-5p	*Ae. aegypti*, *Cx. quinquefasciatus* and *An. gambiae* (Behura et al., 2011)
miR-133-3p	*Ae. aegypti*, *Cx. quinquefasciatus* and *An. gambiae* (Behura et al., 2011)
miR-14-3p	*Ae. aegypti*, *Cx. quinquefasciatus* and *An. gambiae* (Behura et al., 2011), *An. sinensis* (Feng et al., 2018a)
miR-143	*Ae. aegypti* saliva (Maharaj et al., 2015)
miR-184-3p	*Ae. albopictus*, *Cx. quinquefasciatus*, *Ae. albopictus* cell, *An. gambiae*, *Ae. aegypti*, nucleus and the cytoplasm of *Ae. aegypti* cells, *An. stephensi*, *An. sinensis* (Biryukova et al., 2014; Mayoral et al., 2014; Shrinet et al., 2014; Hu et al., 2015; Su et al., 2017; Feng et al., 2018a; Nouzova et al., 2018)
miR-1889-5p	nucleus of *Wolbachia* infected *Ae. aegypti* cells, nucleus and cytoplasm of *Ae. aegypti* cell (Mayoral et al., 2014)
miR-1891-5p	*An. sinensis* (Feng et al., 2018a)
miR-210-3p	*Ae. aegypti*, *Cx. quinquefasciatus* and *An. gambiae* (Behura et al., 2011)
miR-263a-5p/-3p	*Ae. aegypti* and *An. stephensi* (Hu et al., 2015), *An. gambiae* (Biryukova et al., 2014)
miR-263b-5p/-3p	*Ae. aegypti* and *An. stephensi* (Hu et al., 2015)
miR-275-3p/-5p	nucleus and cytoplasm of *Ae. aegypti* cells infected or uninfected with *Wolbachia* (Mayoral et al., 2014) *Ae. albopictus* midgut (Su et al., 2017), *An. sinensis* (Feng et al., 2018a)
miR-276-3p	*An. sinensis* (Feng et al., 2018a), nucleus and the cytoplasm of *Ae. aegypti* cells (Mayoral et al., 2014), *An. gambiae* (Bryant et al., 2019)
miR-277-3p	*An. sinensis* (Feng et al., 2018a), *An. coluzzii* saliva (Bruno et al., 2019)
miR-278-3p/5p	*Ae. aegypti, Cx. quinquefasciatus* and *An. gambiae* (Behura et al., 2011)
miR-281-3p/-5p	*An. sinensis* (Feng et al., 2018a), *An. gambiae* (Biryukova et al., 2014)
miR-287	*Ae. aegypti*, *Cx. quinquefasciatus* and *An. gambiae* (Behura et al., 2011)
miR-2940-5p	*Ae. albopictus* (Skalsky et al., 2010)
miR-2941-3p	*Ae. aegypti* embryos (Hu et al., 2015)
miR-2943-5p	*Ae. aegypti* and *An. stephensi* embryos (Hu et al., 2015)
miR-2945-3p	*Ae. aegypti* and *An. stephensi* embryos (Hu et al., 2015)
miR-2946-3p	*Ae. aegypti* embryos (Hu et al., 2015)
miR-305-5p	*Ae. aegypti*, *Cx. quinquefasciatus*, and *An. gambiae* (Behura et al., 2011)
miR-306-5p	*Ae. aegypti*, *Cx. quinquefasciatus*, and *An. gambiae* (Behura et al., 2011)
miR-307-3p	*Ae. aegypti*, *Cx. quinquefasciatus*, and *An. gambiae* (Behura et al., 2011)
miR-316-5p	*An. sinensis* (Feng et al., 2018a)
miR-317-3p	*Cx. quinquefasciatus*, *Ae. albopictus* C7/10 cells (Skalsky et al., 2010), nucleus and cytoplasm of *Ae. aegypti* cells infected or uninfected with *Wolbachia* (Mayoral et al., 2014), *Ae. albopictus* midgut (Su et al., 2017)
miR-34-5p	*Ae. aegypti*, *Cx. quinquefasciatus* and *An. gambiae* (Behura et al., 2011)
miR-5	*Ae. aegypti*, *Cx. quinquefasciatus* and *An. gambiae* (Behura et al., 2011)
miR-5706	*Ae. albopictus* (Su et al., 2017)
miR-6	*Ae. aegypti*, *Cx. quinquefasciatus* and *An. gambiae* (Behura et al., 2011)
miR-8-3p	*Ae. aegypti*, *Cx. quinquefasciatus An. gambiae* (Skalsky et al., 2010; Behura et al., 2011; Biryukova et al., 2014), C7/10 cells (Skalsky et al., 2010), *Ae. albopictus* (Su et al., 2017), *An. sinensis* (Feng et al., 2018a)
miR-9c	*An. sinensis* (Feng et al., 2018a)
bantam-3p/-5p	*An. gambiae* (Biryukova et al., 2014; Bryant et al., 2019)
let-7-5p	*An. gambiae* (Winter et al., 2007), *Cx. pipiens* (Meuti et al., 2018)
miR-iab-4-5p	*Ae. aegypti*, *Cx. quinquefasciatus* and *An. gambiae* (Behura et al., 2011)

Here, the term “most highly expressed miRNAs” refers to the miRNAs with the greatest abundance among many miRNAs detected in the same sequencing library. During the exploration of miRNA-based approaches, more attention should be devoted to highly expressed mosquito miRNAs.

**Table 2:** Species-specific miRNAs in mosquitoes.

miRNA names	Study material and references
**Conserved insect miRNAs**	
miR-iab-8-5p	eight arthropod species but not mosquitoes (Jain et al., 2014)
miR-133-5p	eight arthropod species but not mosquitoes (Jain et al., 2014)
miR-190-3p	eight arthropod species but not mosquitoes (Jain et al., 2014)
miR-2944a-5p	insect (Allam et al., 2016)
miR-2944b-5p	insect (Allam et al., 2016)
miR-2779	eight arthropod species but not mosquitoes (Jain et al., 2014)
miR-2796-5p	eight arthropod species but not mosquitoes (Jain et al., 2014)
miR-2796-3p	eight arthropod species but not mosquitoes (Jain et al., 2014)
**Conserved mosquito miRNAs**	
miR-1174-3p	mosquito (Winter et al., 2007; Skalsky et al., 2010), e.g., *An. funestus* (Allam et al., 2016), *An. gambiae* (Winter et al., 2007)
miR-1175-5p/-3p	mosquito e.g., *Ae. albopictus* (Gu et al., 2013), *An. funestus* (Allam et al., 2016), *An. gambiae* (Winter et al., 2007)
miR-137-3p	mosquito (Lucas et al., 2015a; Lucas et al., 2015b)
miR-1890-3p	mosquito (Li et al., 2009; Skalsky et al., 2010; Liu et al., 2015; Lucas et al., 2015b), e.g., *Ae. albopictus* (Gu et al., 2013), *Ae. aegypti* (Lucas et al., 2015b)
miR-1891-5p	mosquito (Skalsky et al., 2010), e.g., *Ae. albopictus* (Gu et al., 2013; Liu et al., 2015)
miR-210-3p	mosquito, e.g., *An. funestus* (Allam et al., 2016) and *Ae. albopictus* (Gu et al., 2013)
miR-278-3p	mosquito, e.g., *An. funestus* (Allam et al., 2016)
miR-275-3p	mosquito, e.g., *An. funestus* (Allam et al., 2016)
miR-2941-3p	mosquito (Allam et al., 2016), e.g., *Ae. albopictus* (Gu et al., 2013), *Ae. aegypti* and *An. stephensi* (Hu et al., 2015), insect
miR-2942-3p	mosquito (Allam et al., 2016)
miR-2943-5p	insect (Allam et al., 2016), mosquito (Skalsky et al., 2010; Gu et al., 2013), *Ae. albopictus* (Gu et al., 2013), *Ae. aegypti* and *An. stephensi* (Hu et al., 2015)
miR-2945-3p	mosquitoes (Skalsky et al., 2010)
miR-2946-3p	*Aedes* and *Culex* spp (Gu et al., 2013)., *Ae. aegypti*, *An. stephensi* (Hu et al., 2015), *Ae. albopictus* (Gu et al., 2013)
miR-305-5p	mosquito, e.g., *An. funestus* (Allam et al., 2016)
miR-307-3p	mosquito, e.g., *An. funestus* (Allam et al., 2016)
miR-315–5p	mosquito (Lucas et al., 2015a; Lucas et al., 2015b)
miR-34–3p	mosquito (Lucas et al., 2015a; Lucas et al., 2015b)
miR-989-3p	mosquito, e.g., *An. gambiae* (Winter et al., 2007)
**Conserved miRNAs in different subspecies**	
miR-1889-3p/-5p	*Aedes* and *Culex* spp (Li et al., 2009; Gu et al., 2013; Liu et al., 2015).
miR-282-5p	mosquito, e.g., *Ae. aegypti* and *An. gambiae* but not in *Cx. quinquefasciatus* (Gu et al., 2013)
miR-2940-5p	*Aedes* and *Culex* spp (Gu et al., 2013).
miR-2952	*Cx. quinquefasciatus* (Skalsky et al., 2010)
miR-927-5p	mosquito, e.g., *An. funestus* (Allam et al., 2016), *Ae. aegypti* and *An. gambiae* (but not *Cx. quinquefasciatus*) (Gu et al., 2013)
miR-971-3p	*An. stephensi* (Jain et al., 2014; Hu et al., 2015) [not in *Ae. albopictus* (Su et al., 2017)]

The term “specific” here indicates that the miRNA is restricted to the corresponding mosquito subject. During the exploration of miRNA-based approaches, special attention should be devoted to mosquito-specific miRNAs. Species-specific miRNA may be chosen to establish miRNA-based approaches, and for different mosquito species, the candidate miRNAs for study may differ.

**Table 3:** Sex-specific miRNAs in adult mosquitoes.

Female-specific or enriched miRNAs	Study material and reference	Male-specific or enriched miRNAs	Study material and reference
miR-100-5p	*An. stephensi* (Jain et al., 2015)	miR-1-5p	*An. stephensi* (Jain et al., 2015)
miR-10357-5p	*An. coluzzii* (Bruno et al., 2019)	miR-100-5p	*An. anthropophagus* (Liu et al., 2014)
miR-10358-5p	*An. coluzzii* (Bruno et al., 2019)	miR-1000-5p	*An. anthropophagus* (Liu et al., 2014)
miR-10359-5p	*An. coluzzii* (Bruno et al., 2019)	miR-10381	*An. coluzzii* (Bruno et al., 2019)
miR-10359-3p	*An. coluzzii* (Bruno et al., 2019)	miR-124-3p	*An. anthropophagus* (Liu et al., 2014)
miR-10360-5p	*An. coluzzii* (Bruno et al., 2019)	miR-125-5p	*An. anthropophagus* (Liu et al., 2014)
miR-10362-5p	*An. coluzzii* (Bruno et al., 2019)	miR-125-3p	*An. anthropophagus* (Liu et al., 2014)
miR-10371-5p	*An. coluzzii* (Bruno et al., 2019)	miR-137-3p	*An. anthropophagus* (Liu et al., 2014)
miR-11-3p	*An. stephensi* (Jain et al., 2015)	miR-184-5p	*An. anthropophagus* (Liu et al., 2014)
miR-1174-3p	*An. coluzzii* (Bruno et al., 2019)	miR-1891-5p	*Ae. aegypti*, *An. stephensi* (Hu et al., 2015)
miR-1174-5p	*An. coluzzii* (Bruno et al., 2019)	miR-193-3p	*An. anthropophagus* (Liu et al., 2014)
miR-1175-5p	*An. stephensi* (Jain et al., 2015)	miR-219-5p	*An. coluzzii* (Bruno et al., 2019)
miR-1175-3p	*An. anthropophagus* (Liu et al., 2014), *An. coluzzii* (Bruno et al., 2019)	miR-2765-5p	*An. stephensi* (Jain et al., 2015; Allam et al., 2016)
miR-283-5p	*An. coluzzii* (Bruno et al., 2019)	miR-277-3p	*An. anthropophagus* (Liu et al., 2014)
miR-307-3p	*An. anthropophagus* (Liu et al., 2014)	miR-282-5p	*An. anthropophagus* (Liu et al., 2014)
miR-305-5p	*An. funestus* (Allam et al., 2016)	miR-7-5p	*An. anthropophagus* (Liu et al., 2014), *An. stephensi* (Jain et al., 2015)
miR-315-5p	*An. anthropophagus* (Liu et al., 2014)	miR-981-3p	*An. anthropophagus* (Liu et al., 2014), *An. coluzzii* (Bruno et al., 2019)
miR-79-5p	*An. Anthropophagus* (Liu et al., 2014)	*—*	*—*
miR-929-5p	*An. anthropophagus* (Liu et al., 2014)	*—*	*—*
miR-980-3p	*An. coluzzii* (Bruno et al., 2019)	—	—
miR-988-3p	*An. anthropophagus* (Liu et al., 2014), *An. coluzzii* (Bruno et al., 2019)	*—*	*—*
miR-989-3p	*An. anthropophagus* (Liu et al., 2014), *An. stephensi* (Jain et al., 2015; Allam et al., 2016), *Ae. aegypti* (Allam et al., 2016), *An. coluzzii* (Bruno et al., 2019)	*—*	*—*
miR-989-5p	*An. coluzzii* (Bruno et al., 2019)	—	—

The term “specific” here indicates that miRNA is restricted to one sex or is more abundant in one sex than in the other sex. The symbol “—” indicates that no related evidence is available regarding the corresponding sex of the mosquito.

**Table 4:** Stage- or status-specific miRNAs.

miRNA names	Study materials and expression levels in certain stages or statuses*									
	Egg^&^	Larva^&^			Pupa^&^		Adult^&^		Blood feeding^#^	Other stages^$^
miR-1-3p	***—***	**—**			***An. stephensi*** (Jain et al., 2015)		**—**		***Ae. aegypti* fat body 24 h PBM** (Zhang et al., 2017)**, *Ae. aegypti*** (Hussain et al., 2013), ***An. stephensi*** (Jain et al., 2014), *An. anthropophagus* midgut (Liu et al., 2017)	***Ae. albopictus* nondiapause pharate larva** (Batz et al., 2017), *Cx. pipiens* DR-strain (Hong et al., 2014)
miR-1-5p	**—**	**—**			***An. stephensi*** (Jain et al., 2015)		***An. stephensi*** (Jain et al., 2015)		**—**	**—**
miR-10-3p	**—**	**—**			**—**		**—**		*An. gambiae* (Fu et al., 2017)	**—**
miR-10-5p	***An. sinensis*** (Feng et al., 2018b)	**—**			**—**		***An. stephensi*** (Jain et al., 2015)		**—**	**—**
miR-100-5p	**—**	**—**			**—**		***An. stephensi*** (Jain et al., 2015)		***Ae. aegypti at* 12* h* PBM** (Bryant et al., 2010), *An. anthropophagus* midguts (Liu et al., 2017)	*Cx. pipiens* DR strain (Hong et al., 2014)
miR-1000-5p	**—**	**—**			**—**		**—**		***An. gambiae*** (Biryukova et al., 2014), ***An. anthropophagus* midguts** (Liu et al., 2017), ***Ae. aegypti* fat body at 48 h PBM** (Zhang et al., 2017)	**—**
miR-11-3p	**—**	**—**			**—**		***An. stephensi*** (Jain et al., 2015)**, *An. sinensis*** (Feng et al., 2018b)		***Ae. aegypti* midgut** (Li et al., 2009), ***An. stephensi*** (Jain et al., 2014), ***An. anthropophagus*** (Liu et al., 2017), ***Ae. aegypti* fat body 72 h PBM** (Zhang et al., 2017)	*Cx. pipiens* DR strain (Hong et al., 2014)
miR-11-5p	**—**	**—**			**—**		**—**		***Ae. aegypti* fat body at 48 h PBM** (Zhang et al., 2017)	**—**
miR-1174-5p	**—**	**—**			**—**		**—**		**—**	*Cx. pipiens* DR strain (Hong et al., 2014)
miR-1174-3p	**—**	***An. sinensis*** (Feng et al., 2018b) ***An. stephensi*** (Jain et al., 2015)			**—**		**—**		***An. gambiae* midguts** (Winter et al., 2007), ***Ae. aegypti* and *An. gambiae*** (Jain et al., 2014), ***Ae. aegypti* fat body at 48 h PBM** (Zhang et al., 2017), *An. gambiae* (Fu et al., 2017), *An. anthropophagus* midguts (Liu et al., 2017), *Ae. albopictus* midguts (Su et al., 2017)	**—**
miR-1175-5p	**—**	***An. funestus*** (Allam et al., 2016) ***An. sinensis*** (Feng et al., 2018b)			**—**		***An. sinensis*** (Feng et al., 2018b)		*Ae. albopictus* midguts (Su et al., 2017)	*Cx. pipiens* DR strain (Hong et al., 2014)
miR-1175-3p	**—**	***An. sinensis*** (Feng et al., 2018b)			**—**		***An. sinensis*** (Feng et al., 2018b)		***An. gambiae* midgut** (Winter et al., 2007), ***Aedes* spp.** (Jain et al., 2014),**** *Ae. albopictus* midgut (Su et al., 2017)	**—**
miR-12-5p	**—**	**—**			**—**		**—**		**leftover of *An. gambiae*** (Winter et al., 2007), *An. anthropophagus* midguts (Liu et al., 2017), *Ae. albopictus* midguts (Su et al., 2017)	**—**
miR-124-3p	***An. sinensis*** (Feng et al., 2018b)	**—**			**—**		**—**		**—**	old *Cx. pipiens* (Meuti et al., 2018)
miR-125-5p	**—**	***An. funestus*** (Allam et al., 2016)			**—**		**—**		***Ae. aegypti* 12* h* PBM** (Bryant et al., 2010), ***An. anthropophagus* midguts** (Liu et al., 2017), ***Ae. aegypti* fat body at 72 h PBM** (Zhang et al., 2017)	*Cx. pipiens* DR strain (Hong et al., 2014)
miR-127	**—**	**—**			**—**		**—**		**—**	*Cx. pipiens* DR strain (Hong et al., 2014)
miR-13-3p	**—**	**—**			**—**		**—**		*An. anthropophagus* midguts (Liu et al., 2017)	*Cx. pipiens* DR strain (Hong et al., 2014)
miR-13-5p	**—**	**—**			**—**		**—**		***Ae. aegypti* fat body at 48 h PBM** (Zhang et al., 2017)	
miR-133-3p	***An. funestus*** (Allam et al., 2016)	***An. funestus*** (Allam et al., 2016)			***An. stephensi*** (Jain et al., 2015)		**—**		***Ae. aegypti* fat body at 48 h PBM** (Zhang et al., 2017)	*Cx. pipiens* DR strain (Hong et al., 2014)
miR-133-5p	**—**	**—**			***An. stephensi*** (Jain et al., 2015)		***An. stephensi*** (Jain et al., 2015)		**—**	**—**
miR-137-3p	**—**	**—**			**—**		**—**		*An. anthropophagus* midguts (Liu et al., 2017)	**—**
miR-137-5p	***An. funestus*** (Allam et al., 2016)	***An. funestus*** (Allam et al., 2016)			**—**		**—**		**—**	**—**
miR-14-5p	**—**	**—**			**—**		**—**		**—**	***Ae. albopictus* nondiapause pharate larva** (Batz et al., 2017)
miR-14-3p	***An. funestus*** (Allam et al., 2016)	***An. funestus*** (Allam et al., 2016)			**—**		**—**		***Ae. aegypti*** (Hussain et al., 2013), *An. anthropophagus* midguts (Liu et al., 2017)	old or DR *Cx. pipiens* (Hong et al., 2014; Meuti et al., 2018)
miR-1767	**—**	**—**			**—**		**—**		**midguts of *Ae. albopictus*** (Su et al., 2017)	**—**
miR-184-3p	***An. funestus*** (Allam et al., 2016)	**—**			**—**		**—**		***Ae. aegypti*** (Li et al., 2009), ***Ae. aegypti* fat body at 72 h PBM** (Zhang et al., 2017), fat body of *Ae. aegypti* and *An. gambiae* (Fu et al., 2017), *An. anthropophagus* midguts (Liu et al., 2017), *Ae. albopictus* midguts (Su et al., 2017), *Aedes aegypti* (Nouzova et al., 2018)	**—**
miR-1889-5p	**—**	**—**			**—**		**—**		***Ae. aegypti* fat body at 36 h PBM** (Zhang et al., 2017)	**—**
miR-1890-3p	**—**	**—**			***Ae. aegypti*, *An. stephensi*** (Hu et al., 2015; Jain et al., 2015; Allam et al., 2016; Batz et al., 2017)		**—**		***Ae. aegypti* fat body at 36 h PBM** (Zhang et al., 2017)	***Ae. albopictus* diapause oocyte** (Batz et al., 2017), *Cx. pipiens* DR strain (Hong et al., 2014)
miR-1891-5p	**—**	**—**			***An. sinensis*** (Feng et al., 2018b)		***An. stephensi*** (Jain et al., 2015)		**—**	*Cx. pipiens* DR strain (Hong et al., 2014)
miR-190-5p	**—**	**—**							***Ae. aegypti* midgut** (Li et al., 2009), *An. Stephensi* (Jain et al., 2014)	*Cx. pipiens* DR strain (Hong et al., 2014)
miR-190-3p	**—**	**—**			***An. stephensi*** (Jain et al., 2015)		***An. stephensi*** (Jain et al., 2015)		*An. Stephensi* (Jain et al., 2014)	**—**
miR-193-5p	**—**	**—**							***Ae. albopictus* midguts** (Su et al., 2017)	**—**
miR-193-3p	**—**	**—**			***An. stephensi*** (Jain et al., 2015) ***An. funestus*** (Allam et al., 2016) ***An. sinensis*** (Feng et al., 2018b)		**—**		**—**	**—**
miR-1951	**—**	**—**			**—**		**—**		***Ae. albopictus* midguts** (Su et al., 2019)	**—**
miR-210-3p	***An. stephensi*** (Mead and Tu, 2008)						***An. stephensi*** (Mead and Tu, 2008; Jain et al., 2015)		***Ae. aegypti* fat body at 48 h PBM** (Zhang et al., 2017)	***Cx. pipiens* DR strain** (Hong et al., 2014)
miR-219-5p	**—**	**—**			**—**		***An. stephensi*** (Jain et al., 2015)		**—**	**—**
miR-2491-3p	**—**	**—**			***An. sinensis*** (Feng et al., 2018b)		**—**		**—**	**—**
miR-252-5p	**—**	**—**			**—**		**—**		***Ae. aegypti* fat body at 6 h PBM** (Zhang et al., 2017)	*Cx. pipiens* DR strain (Hong et al., 2014)
miR-252-3p	**—**	**—**			**—**		**—**		***Ae. aegypti* fat body at 6 h PBM** (Zhang et al., 2017)	
miR-263a-5p	**—**	**—**			**—**		**—**			*Cx. pipiens* DR strain (Hong et al., 2014)
miR-275-3p	**—**	**—**			**—**		**—**		***An. stephensi*** (Jain et al., 2014), ***An. anthropophagus* midguts** (Liu et al., 2017), ***Ae. aegypti* fat body at 48h PBM** (Zhang et al., 2017), ***An. gambiae*** (Lampe and Levashina, 2018), *Ae.albopictus* midgut (Su et al., 2017)	old *Cx. pipiens* (Meuti et al., 2018), *Cx. pipiens* DR-strain (Hong et al., 2014)
miR-275-5p	**—**	**—**			**—**		**—**		*Ae. aegypti* fat body at 24 h PBM (Zhang et al., 2017)	
miR-276-3p	**—**	**—**			**—**		**—**		*An.anthropophagus* midgut (Liu et al., 2017), *Ae.aegypti* fat body at 24h PBM (Zhang et al., 2017)	*Cx. pipiens* DR strain (Hong et al., 2014)
miR-276-5p	**—**	**—**			**—**		**—**		***Ae. aegypti* fat body at 24 h PBM** (Zhang et al., 2017), ***An. gambiae* midgut** (Lampe and Levashina, 2018), *An. gambiae* head (Lampe and Levashina, 2018)	
miR-2765-5p	**—**	**—**			**—**		***An. stephensi*** (Jain et al., 2015) *An. funestus* (Allam et al., 2016)		**—**	**—**
miR-277-3p				***An. funestus*** (Allam et al., 2016)		***An. stephensi*** (Jain et al., 2015)		***An. stephensi*** (Jain et al., 2015)	*An. anthropophagus* midguts (Liu et al., 2017)	old or DR *Cx. pipiens* (Hong et al., 2014; Meuti et al., 2018)
miR-2779	**—**			**—**		**—**			**—**	**—**
miR-278-3p				***An. funestus*** (Allam et al., 2016)					***Ae.aegypti* fat body at 48h PBM** (Zhang et al., 2017), *An.anthropophagus* midgut (Liu et al., 2017)	*Cx. pipiens* DR strain (Hong et al., 2014)
miR-278-5p	**—**			**—**		**—**		**—**	***Ae. aegypti* fat body at 6 h PBM** (Zhang et al., 2017), fat body of *Ae. aegypti* (Fu et al., 2017)	
miR-279-3p	***An. gambiae*** (Allam et al., 2016)			***An. gambiae*** (Allam et al., 2016)		**—**		**—**	***Ae.aegypti* fat body at 24h PBM** (Zhang et al., 2017), *An.anthropophagus* midgut (Liu et al., 2017)	*Cx. pipiens* DR strain (Hong et al., 2014)
miR-2796-3p	***An. funestus*** (Allam et al., 2016)			**—**		**—**		**—**	**—**	**—**
miR-281-3p				***An. sinensis*** (Feng et al., 2018b)				***An. sinensis*** (Feng et al., 2018b)	***An. stephensi*** (Jain et al., 2014), ***Ae. aegypti* fat body at 48 h PBM** (Zhang et al., 2017)	***Cx. pipiens* DR strain** (Hong et al., 2014)
miR-281-5p	***An. gambiae*** (Allam et al., 2016)			***An. gambiae*** (Allam et al., 2016)				***An. sinensis*** (Feng et al., 2018b)	***An. stephensi*** (Jain et al., 2014), ***Ae. aegypti* fat body at 48 h PBM** (Zhang et al., 2017), *Ae. aegypti* (Li et al., 2009), *Ae. albopictus* midgut (Su et al., 2017)	
miR-282-5p	**—**			**—**				***An. stephensi*** (Jain et al., 2015)	***—***	***Ae. albopictus* diapause pharate larva** (Batz et al., 2017)
miR-283-5p				***Ae. albopictus*** (Batz et al., 2017)				***—***	*Ae. albopictus* midguts (Su et al., 2017)	
miR-2840	**—**			**—**		**—**		**—**	**—**	***Cx. pipiens* DR strain** (Hong et al., 2014)
miR-285-3p	**—**			**—**		***An. stephensi*** (Jain et al., 2015)		**—**	**—**	*Cx. pipiens* DR strain (Hong et al., 2014)
miR-286-3p	**—**			**—**		***An. stephensi*** (Jain et al., 2015)			fat body of *Ae. aegypti* (Fu et al., 2017)	
miR-2940-3p	**—**			**—**		**—**		**—**	***Ae. aegypti* fat body at 36 h PBM** (Zhang et al., 2017)	
miR-2940-5p	**—**			**—**		**—**		**—**	***Ae. aegypti*** (Hussain et al., 2013)	
miR-2941-3p	**—**			**—**		**—**		**—**	***Ae. aegypti* fat body at 48 h PBM** (Zhang et al., 2017), *Ae. albopictus* midguts (Su et al., 2017)	***Cx. pipiens* DR strain** (Hong et al., 2014)
miR-2942-3p	***—***			***Ae. albopictus*** (Puthiyakunnon et al., 2013)		**—**		**—**	**—**	***Ae. albopictus* nondiapause pharate larva** (Batz et al., 2017), *Cx. pipiens* DR strain (Hong et al., 2014)
miR-2943-5p	***An.anthropophagus*** (Liu et al., 2014) ***An. sinensis*** (Feng et al., 2018b)			**—**		**—**		**—**	**—**	**—**
miR-2944a-5p	**—**			**—**		**—**		**—**	***An. gambiae*** (Fu et al., 2017)	***—***
miR-2945-3p	**—**			**—**		**—**		**—**	***Ae. aegypti* fat body at 36 h PBM** (Zhang et al., 2017)	*—*
miR-2946-3p	**—**			**—**		**—**		**—**	***Ae. aegypti* fat body at 48 h PBM** (Zhang et al., 2017)	***—***
miR-2951-5p	**—**			**—**		**—**		**—**	**midgut of *Ae. albopictus*** (Su et al., 2017)	*Cx. pipiens* DR strain (Hong et al., 2014)
miR-2952	**—**			**—**		**—**		**—**	**—**	***Cx. pipiens* DR strain** (Hong et al., 2014)
miR-2981	**—**		**—**		**—**		**—**		**—**	*Cx. pipiens* DR strain (Hong et al., 2014)
miR-2c-3p	**—**		**—**		**—**		**—**		**—**	*Cx. pipiens* DR strain (Hong et al., 2014; Guo et al., 2017)
miR-309a-3p	***An. funestus*** (Hu et al., 2015; Allam et al., 2016) ***Ae. aegypti*, *An. stephensi*** (Hu et al., 2015)				***An. stephensi*** (Jain et al., 2015)				***—***	old *Cx. pipiens* (Meuti et al., 2018), ***Cx. pipiens* DR strain** (Hong et al., 2014)
miR-305-5p	***—***		***An. funestus*** (Allam et al., 2016)		***An. funestus*** (Allam et al., 2016)		***An. funestus*** (Allam et al., 2016)		***Ae. aegypti*** (Bryant et al., 2010), ***An. Stephensi*** (Jain et al., 2014), ***Ae.aegypti* fat body at 48 h PBM** (Zhang et al., 2017), ***An. gambiae* midgut** (Lampe and Levashina, 2018), *An.anthropophagus* midgut (Liu et al., 2017)	*Cx. pipiens* DR strain (Hong et al., 2014)
miR-305-3p	**—**		**—**		**—**		**—**		*Ae. aegypti* fat body at 24 h PBM (Zhang et al., 2017)	**nondiapausing *Cx. pipiens*** (Meuti et al., 2018)
miR-306-5p	**—**		**—**		**—**		**—**		***Ae. aegypti*** (Li et al., 2009), ***An. stephensi*** (Jain et al., 2014), ***Ae. aegypti* fat body at 72 h PBM** (Zhang et al., 2017), ***An. gambiae* ovary** (Nouzova et al., 2018), *An.anthropophagus* midgut (Liu et al., 2017)	***Cx. pipiens* DR strain** (Hong et al., 2014)
miR-306-3p	**—**		**—**		**—**		**—**		*Ae. aegypti* fat body at 72 h PBM (Zhang et al., 2017)	***—***
miR-307-3p	**—**		**—**		**—**		**—**		*An. anthropophagus* midguts (Liu et al., 2017)	*Cx. pipiens* DR strain (Hong et al., 2014)
miR-308-3p	**—**		**—**		**—**		**—**		***An. gambiae*** (Biryukova et al., 2014), ***Ae. aegypti* fat body at 24h PBM** (Zhang et al., 2017)	*—*
miR-308-5p	**—**		**—**		**—**		**—**		***Ae. aegypti* fat body at 72 h PBM** (Zhang et al., 2017)	***—***
miR-315-5p	**—**		**—**		*An. stephensi* (Jain et al., 2015)				**midgut of *An. anthropophagus*** (Liu et al., 2017)	*—*
miR-315-3p	**—**		**—**		**—**		**—**		**midgut of *An. anthropophagus*** (Liu et al., 2017)	*—*
miR-316-5p	***An. funestus*** (Allam et al., 2016)		***An. funestus*** (Allam et al., 2016)		**—**		**—**		***Ae. aegypti* fat body at 48 h PBM** (Zhang et al., 2017)	***Cx. pipiens* DR strain** (Hong et al., 2014)
miR-317-3p	***—***		***An. stephensi*** (Jain et al., 2015) ***An. funestus*** (Allam et al., 2016) ***An. sinensis*** (Feng et al., 2018b)				***An. stephensi*** (Jain et al., 2015) ***An. funestus*** (Allam et al., 2016) ***An. sinensis*** (Feng et al., 2018b)		***An. gambiae* midguts** (Winter et al., 2007), ***Ae. aegypti* midguts** (Li et al., 2009; Su et al., 2017), ***Ae. aegypti*** (Hussain et al., 2013; Nouzova et al., 2018), ***Ae. aegypti* fat body at 36 h PBM** (Zhang et al., 2017), *Ae. albopictus* midguts (Su et al., 2017)	***Cx. pipiens* DR strain** (Hong et al., 2014)
miR-33-5p	**—**		**—**		**—**		**—**		**—**	*Cx. pipiens* DR strain (Hong et al., 2014)
miR-34-3p	**—**		**—**		**—**		**—**		***Ae. aegypti* fat body at 48 h PBM** (Zhang et al., 2017)	***—***
miR-34-5p	—		***An. stephensi*** (Jain et al., 2015) ***An. funestus*** (Allam et al., 2016) ***An. sinensis*** (Feng et al., 2018b)		**—**		***An. stephensi*** (Jain et al., 2015) ***An. funestus*** (Allam et al., 2016) ***An. sinensis*** (Feng et al., 2018b)		***Ae. aegypti* midgut** (Li et al., 2009; Su et al., 2017), ***Ae. aegypti* fat body at 24 h PBM** (Zhang et al., 2017), *An. anthropophagus* midguts (Liu et al., 2017), *Ae. albopictus* midguts (Su et al., 2017)	***—***
miR-375-3p	***—***		***An. stephensi*** (Jain et al., 2015)		***Ae. aegypti*** (Nouzova et al., 2018)		***An. stephensi*** (Jain et al., 2015)		***Ae. aegypti*** (Hussain et al., 2013), ***Ae. albopictus* midguts** (Su et al., 2017), ***Ae. aegypti* fat body at 48 h PBM** (Zhang et al., 2017), ***Cx. pipiens*** (Meuti et al., 2018)	**nondiapausing *Cx. pipiens*** (Meuti et al., 2018) *Cx. pipiens* DR strain (Hong et al., 2014)
miR-375-5p	**—**		**—**		**—**		**—**		**—**	***Cx. pipiens* DR strain** (Hong et al., 2014)
miR-3809-3p	**—**		**—**		**—**		**—**		***Ae. albopictus* midguts** (Su et al., 2017)	old *Cx. pipiens* (Meuti et al., 2018)
miR-3809-5p	**—**		**—**		**—**		**—**		***Ae. albopictus* midguts** (Su et al., 2017)	*—*
miR-424-3p	**—**		**—**		**—**		**—**		***Ae. albopictus* midguts** (Su et al., 2017)	***—***
miR-4448	**—**		**—**		**—**		**—**		***Ae. albopictus* midguts** (Su et al., 2017)	***Cx. pipiens* DR strain** (Hong et al., 2014)
miR-4728-5p	**—**	**—**			**—**		**—**		***Ae. albopictus* midguts** (Su et al., 2017)	***—***
miR-493-3p	**—**	**—**			**—**		**—**		***—***	***Cx. pipiens* DR strain** (Hong et al., 2014)
miR-4968-3p	***—***	***An. sinensis*** (Feng et al., 2018b)			**—**		**—**		**—**	**—**
miR-622	**—**	**—**			**—**		**—**		***Ae. albopictus* midguts** (Su et al., 2017)	***—***
miR-7-5p	*—*	***An. stephensi*** (Jain et al., 2015)			**—**		**—**		***An. gambiae*** (Biryukova et al., 2014)	*Cx. pipiens* DR strain (Hong et al., 2014)
miR-71-3p	**—**	**—**			**—**		**—**		***Ae. aegypti* fat body at 48 h PBM** (Zhang et al., 2017), *An. anthropophagus* midguts (Liu et al., 2017), *Ae. albopictus* midguts (Su et al., 2017)	*Cx. pipiens* DR strain (Hong et al., 2014)
miR-79-3p	**—**	**—**			**—**		**—**		***Ae. aegypti* fat body at 6 h PBM** (Zhang et al., 2017)	*Cx. pipiens* DR strain (Hong et al., 2014)
miR-79-5p	**—**	**—**			**—**		**—**		*An. anthropophagus* midguts (Liu et al., 2017)	
miR-8-3p	—	***An. funestus*** (Allam et al., 2016)			***Ae. aegypti*** (Bryant et al., 2010)		*—*		***An. anthropophagus* midguts** (Liu et al., 2017)**, *Ae. aegypti* fat body at 24 h PBM** (Zhang et al., 2017), *Ae. aegypti* midguts (Li et al., 2009), *Ae. aegypti* (Bryant et al., 2010), *Ae.albopictus* (Su et al., 2017)	old *Cx. pipiens* (Meuti et al., 2018), *Cx. pipiens* DR strain (Hong et al., 2014)
miR-87-3p	*An. funestus* (Allam et al., 2016)	***—***			*An. funestus* (Allam et al., 2016)		***—***		***Ae. aegypti* fat body at 48 h PBM** (Zhang et al., 2017)	*Cx. pipiens* DR strain (Hong et al., 2014)
miR-927-5p	**—**	**—**			*—*		***An. stephensi*** (Jain et al., 2015)		***Ae. aegypti* fat body at 48 h PBM** (Zhang et al., 2017)	*—*
miR-927-3p	***An. funestus*** (Allam et al., 2016)	***An. funestus*** (Allam et al., 2016)			*—*		***An. stephensi*** (Jain et al., 2015)		**—**	**—**
miR-929-3p	**—**	**—**			**—**		**—**		*An. stephensi* (Jain et al., 2014)	*—*
miR-929-5p	**—**	**—**			**—**		***An. stephensi*** (Jain et al., 2015), *An. funestus* (Allam et al., 2016)		***Ae. aegypti* fat body at 48 h PBM** (Zhang et al., 2017)	*—*
miR-92a-3p	***—***	*An. funestus* (Allam et al., 2016)			**—**		**—**		**—**	**—**
miR-932-5p	**—**	**—**			**—**		**—**		***An. anthropophagus* midguts** (Liu et al., 2017), *An. Stephensi* (Jain et al., 2014)	*Cx. pipiens* DR strain (Hong et al., 2014)
miR-956-3p	**—**	**—**			**—**		**—**		***An. gambiae*** (Biryukova et al., 2014), *Ae.albopictus* and *Ae. aegypti* (Su et al., 2017)	
miR-957-3p	**—**	**—**			**—**		**—**		***An.anthropophagus* midguts** (Liu et al., 2017), ***Ae.aegypti* fat body at 48h PBM** (Zhang et al., 2017)	***Ae. albopictus* diapause oocyte** (Batz et al., 2017), *Cx. pipiens* DR strain (Hong et al., 2014)
miR-965-3p	**—**	**—**			**—**		*An. stephensi* (Jain et al., 2015)		***—***	***Cx. pipiens* DR strain** (Hong et al., 2014)
miR-970-3p	***An. funestus*** (Allam et al., 2016)	***An. funestus*** (Allam et al., 2016)			**—**		**—**		***Ae. aegypti* fat body at 36h PBM** (Zhang et al., 2017), *An.anthropophagus* midgut (Liu et al., 2017)	*Cx. pipiens* DR strain (Hong et al., 2014)
miR-976-5p	**—**	**—**			**—**		**—**		***Ae. albopictus* and *Ae. aegypti*** (Su et al., 2017)	***—***
miR-980-3p	**—**	**—**			**—**		***An. stephensi*** (Jain et al., 2015)		**—**	**—**
miR-981-3p	**—**	**—**			**—**		**—**		***An. anthropophagus* gut (** Liu et al., 2017), ***Ae.aegypti* fat body at 48h PBM** (Zhang et al., 2017)	*Cx. pipiens* DR strain (Hong et al., 2014)
miR-988-5p	**—**	**—**			***An. stephensi*** (Jain et al., 2015)		***An. stephensi*** (Jain et al., 2015)		***Ae. aegypti* fat body at 72 h PBM** (Zhang et al., 2017)	*—*
miR-988-3p	**—**	**—**			**—**		**—**		*An. gambiae* (Fu et al., 2017)	*—*
miR-989-3p	**—**	**—**			**—**		***An. stephensi*** (Jain et al., 2015)		***Ae. aegypti*** (Li et al., 2009), ***An. stephensi*** (Jain et al., 2014), ***An. anthropophagus* midguts** (Liu et al., 2017), ***Ae. aegypti* fat body at 24 h PBM** (Zhang et al., 2017), *Ae. albopictus* (Su et al., 2017), *An. gambiae* ovaries (Lampe and Levashina, 2018)	***Cx. pipiens* DR strain** (Hong et al., 2014)
miR-993-5p	**—**	**—**			**—**		***An. stephensi*** (Jain et al., 2015)		**—**	**—**
miR-993-3p	***An. funestus*** (Allam et al., 2016)	***An. funestus*** (Allam et al., 2016)			**—**		**—**		***Ae. aegypti* fat body at 36 h PBM** (Zhang et al., 2017)	*Cx. pipiens* DR strain (Hong et al., 2014)
miR-996-3p	***An. funestus*** (Allam et al., 2016)	***An. stephensi*** (Jain et al., 2015) ***An. funestus*** (Allam et al., 2016)			*—*		***An. stephensi*** (Jain et al., 2015)		***Ae. aegypti* fat body at 72 h PBM** (Zhang et al., 2017), *An. anthropophagus* midgut (Liu et al., 2017)	***—***
miR-996-5p	**—**	**—**			**—**		**—**		*An. anthropophagus* midgut (Liu et al., 2017)	***—***
miR-988-3p	**—**	**—**			**—**		**—**		***Ae. aegypti*** (Li et al., 2009), ***Ae. aegypti* fat body at 72 h PBM** (Zhang et al., 2017)	
miR-998-3p	***An. funestus*** (Allam et al., 2016)	***—***			***An. stephensi*** (Jain et al., 2015)		***—***		*Ae. albopictus* (Su et al., 2017)	*Cx. pipiens* DR strain (Hong et al., 2014)
miR-999-3p	**—**	**—**			**—**		**—**		***Ae. aegypti* fat body at 24 h PBM** (Zhang et al., 2017)	*Cx. pipiens* DR strain (Hong et al., 2014)
miR-9a-5p	**—**	**—**			**—**		**—**		***—***	*Cx. pipiens* DR strain (Hong et al., 2014)
miR-iab-4-3p	**—**	**—**			***An. stephensi*** (Jain et al., 2015)				**—**	**—**
miR-iab-4-5p	**—**	**—**			**—**		**—**		***Ae. aegypti* fat body at 72 h PBM** (Zhang et al., 2017)	
bantam-3p	**—**	**—**			***An. stephensi*** (Jain et al., 2015)		*—*		***Ae. aegypti* fat body at 6h PBM** (Zhang et al., 2017), *An.anthropophagus* midgut (Liu et al., 2017)	***Ae.albopictus* oocyte** (Batz et al., 2017)
bantam-5p	**—**	**—**			***Ae. aegypti*** (Bryant et al., 2010)		*—*		***Ae. aegypti*** (Hussain et al., 2013)	***—***
let-7-5p	*An. funestus* (Mead and Tu, 2008; Allam et al., 2016)	***An. funestus*** (Allam et al., 2016)			***Ae. albopictus*** (Gu et al., 2013)**, *An. stephensi*** (Jain et al., 2015)		***—***		***Ae. aegypti* gut at 12 h PBM** (Bryant et al., 2010), ***Ae. aegypti* fat body at 6 h PBM** (Zhang et al., 2017), *An. anthropophagus* midguts (Liu et al., 2017), *Ae. albopictus* (Su et al., 2017)	***—***

^*^The “study materials” are written in two styles, namely **bold** and nonbold, which indicates that the miRNAs are upregulated and downregulated, respectively. “—”, no evidence of upregulation or downregulation is available. ^&^The term “specific” here indicates that miRNAs are upregulated or downregulated in one mosquito development stage when compared with the others. ^**#**^The term “specific” here indicates that the miRNA is upregulated or downregulated in blood-feeding mosquitoes compared with non-blood-feeding mosquitoes, in one study (Zhang et al., 2017), the comparisons were conducted at the time points 72 post eclosion, 6, 12, 24, 36, 48 and 72h post blood meal (PBM). ^$^The term “specific” here indicates that the miRNA is upregulated or downregulated in one group compared with the opposite group. DR, deltamethrin-resistant.

**Table 5:** Tissue-, organ- or cell compartment- specific miRNAs.

miRNA names	Study materials and enriched tissues, organs, or cell compartments							
	Ovary^&^	Salivary glands^&^	Midgut^&^	Brain^&^	Fat body^&^	Thorax^&^	Cell cytoplasm^#^	Cell nucleus^#^
miR-1-3p	***—***	***—***	***—***	***—***	***—***	***—***	infected cells (Mayoral et al., 2014)	*Ae. aegypti* cells (Mayoral et al., 2014)
miR-10-3p	*An. gambiae* (Nouzova et al., 2018)	***—***	***—***	***—***	***—***	***—***	***—***	***—***
miR-10-5p	*An. gambiae* (Lampe and Levashina, 2018)	***—***	***—***	***—***	***—***	***—***	*Ae. aegypti* cells (Mayoral et al., 2014)	*—*
miR-100-5p	***—***	***—***	***—***	***—***	***—***	***—***	***—***	***—***
miR-10355-3p	*An. gambiae* (Bryant et al., 2019)	*—*	*—*	*—*	*—*	*—*	***—***	***—***
miR-10355-5p	*An. gambiae* (Bryant et al., 2019)	***—***	***—***	***—***	***—***	***—***	***—***	***—***
miR-10365-5p	*An. gambiae* (Bryant et al., 2019)	***—***	***—***	***—***	***—***	***—***	***—***	***—***
miR-10367-5p	*An. gambiae* (Bryant et al., 2019)	***—***	***—***	***—***	***—***	***—***	***—***	***—***
miR-10368-3p	*An. gambiae* (Bryant et al., 2019)	*—*	*—*	*—*	*—*	*—*	***—***	***—***
miR-10365-3p	***—***	*An. coluzzii* (Bruno et al., 2019)	*—*	*—*	*—*	*—*	***—***	***—***
miR-10376-3p	***—***	*—*	***—***	***—***	***—***	***—***	***—***	***—***
miR-11-3p	***—***	*An. coluzzii* (Bruno et al., 2019)	***—***	***—***	***—***	***—***	***—***	***—***
miR-1174-3p	***—***	***—***	***—***	*An. gambiae* (Bryant et al., 2020)	***—***	***—***	***—***	***—***
miR-1174-5p	*—*	***—***	***—***	***—***	***—***	***—***	***—***	***—***
miR-1175-5p	*An. gambiae* (Bryant et al., 2019)	***—***	***—***	***—***	***—***	***—***	*Ae. aegypti* cells (Mayoral et al., 2014)	***—***
miR-1175-3p	*An. gambiae* (Bryant et al., 2019)	***—***	***—***	***—***	***—***	***—***	*Ae. aegypti* cells (Mayoral et al., 2014)	***—***
miR-12-5p	*An. gambiae* (Bryant et al., 2019)	*An. coluzzii* (Bruno et al., 2019)	*An. gambiae* (Winter et al., 2007; Bryant et al., 2019)	*—*	*—*	*—*	*—*	*Ae. aegypti* cells (Mayoral et al., 2014)
miR-12-3p	*An. gambiae* (Bryant et al., 2019)	*An. coluzzii* (Bruno et al., 2019)	*An. gambiae* (Winter et al., 2007; Bryant et al., 2019)	*—*	***—***	***—***	*—*	*Ae. aegypti* cells (Mayoral et al., 2014)
miR-124-3p	*—*	*—*	*—*	*An. gambiae* (Lampe and Levashina, 2018)	***—***	***—***	*—*	*—*
miR-12414-3p	*An. gambiae* (Bryant et al., 2019)	*—*	*—*	*—*	***—***	*—*	*—*	*—*
miR-125-5p		*An. coluzzii* (Bruno et al., 2019)	***—***	***—***	***—***	***—***	*Ae. aegypti* cells (Mayoral et al., 2014)	***—***
miR-133-3p	*—*	*—*	***—***	***—***	***—***	***—***	*—*	*—*
miR-137-3p	*—*	*—*	***—***	***—***	***—***	*—*	*Ae. aegypti* cells (Mayoral et al., 2014)	***—***
miR-14-3p	*—*	*—*	***—***	***—***	*Ae. aegypti* (Bryant et al., 2010)	*—*	*—*	*—*
miR-1889-3p	***—***	*An. coluzzii* (Bruno et al., 2019)	***—***	***—***	***—***	*—*	infected cells (Mayoral et al., 2014)	*Ae. aegypti* cells (Mayoral et al., 2014)
miR-1889-5p	**—**	*An. coluzzii* (Bruno et al., 2019)	***—***	***—***	***—***	*—*	*—*	*—*
miR-1891-3p	*—*	*—*	***—***	***—***	*An. gambiae* (Bryant et al., 2019)	*—*	*—*	***—***
miR-1891-5p	*An. gambiae* (Bryant et al., 2019)	***—***	*An. gambiae* (Bryant et al., 2019)	***—***	*—*	*—*	*—*	***—***
miR-210-3p	*—*	*—*	***—***	*An. gambiae* (Lampe and Levashina, 2018)			*Ae. aegypti* cells (Mayoral et al., 2014)	
miR-252-5p	*—*	*—*	***—***	*—*	*—*	***—***	*—*	***—***
miR-252-3p	*—*	*—*	***—***	*—*	*—*	***—***	*—*	***—***
miR-275-3p	*—*	*—*	***—***	*An. gambiae* (Lampe and Levashina, 2018)	*—*	*—*	*—*	***—***
miR-275-5p	***—***	*An. coluzzii* (Bruno et al., 2019)	*—*	*—*	***—***	*—*	*—*	***—***
miR-276-5p	*—*	*—*	***—***	*An. gambiae* (Nouzova et al., 2018; Lampe and Levashina, 2018)	*An. gambiae* (Nouzova et al., 2018)	*—*	*—*	***—***
miR-2765-5p	*An. gambiae* (Bryant et al., 2019)	*—*	*—*	*—*	*—*	***—***	***—***	***—***
miR-277-3p	*—*	*—*	*—*	*—*	***—***	*An. gambiae* (Winter et al., 2007)	***—***	***—***
miR-279-3p	*An. gambiae* (Lampe and Levashina, 2018)	*—*	*—*	*—*	*—*	***—***	*—*	***—***
miR-281-3p	*An. gambiae* (Lampe and Levashina, 2018)	*An. coluzzii* (Bruno et al., 2019)	*An. gambiae* (Nouzova et al., 2018; Bryant et al., 2019)	*An. gambiae* (Lampe and Levashina, 2018)	*—*	*—*	*—*	***—***
miR-281-5p		*An. coluzzii* (Bruno et al., 2019)	*An. gambiae* (Nouzova et al., 2018; Bryant et al., 2019)	*—*	*—*	*—*	*Ae. aegypti* cells (Mayoral et al., 2014)	*Ae. aegypti* cells (Mayoral et al., 2014)
miR-282-5p	*—*	*—*	***—***	*—*	*—*	*—*	*Ae. aegypti* cells (Mayoral et al., 2014)	
miR-283-5p	***—***	*An. coluzzii* (Bruno et al., 2019)	*An. gambiae* (Winter et al., 2007; Bryant et al., 2019)	***—***	***—***	*An. gambiae* (Winter et al., 2007)	*—*	***—***
miR-285-3p	*—*	*—*	*—*	*—*	*—*	***—***		
**miR-286-3p**	*Ae. aegypti* (Akbari et al., 2013)	*—*	*—*	*—*	*—*	***—***		
miR-2945-3p	***—***	***—***	*Ae. albopictus* (Su et al., 2017)	*—*	*—*	***—***	*—*	***—***
miR-2c-3p	*—*	*An. coluzzii* (Bruno et al., 2019)	*—*	*—*	***—***	*—*	*—*	***—***
miR-305-5p	*An. gambiae* (Lampe and Levashina, 2018)	*—*	*—*	*—*	*—*	*—*	*Ae. aegypti* cells (Mayoral et al., 2014)	*Ae. aegypti* cells (Mayoral et al., 2014)
miR-306-5p	*An. gambiae* (Nouzova et al., 2018)	*—*	*—*	*—*	*—*	*—*	*—*	***—***
miR-3069	*—*	*—*	***—***	*—*	*—*	***—***	*—*	***—***
miR-307-3p	***—***	*An. coluzzii* (Bruno et al., 2019)		*An. gambiae* (Lampe and Levashina, 2018)	*An. gambiae* (Lampe and Levashina, 2018)	*—*	*—*	***—***
miR-308-3p	***—***	*An. coluzzii* (Bruno et al., 2019)	*—*	*—*	*—*	*—*	*—*	***—***
miR-309a-3p	*An.gambiae* (Bryant et al., 2019)	*—*	*—*	*—*	*—*	***—***	*—*	***—***
miR-375-5p	***—***	*An. coluzzii* (Bruno et al., 2019)	*—*	*—*	***—***	*—*	*—*	***—***
miR-375-3p	***—***	*An. coluzzii* (Bruno et al., 2019)	*—*	*—*	*—*	***—***	*—*	*—*
miR-7-5p	*—*	*—*	***—***	*An. gambiae* (Lampe and Levashina, 2018)	*—*	*—*	*—*	***—***
miR-71-3p	*—*	*—*	***—***	*—*	*—*	*—*	infected cells (Mayoral et al., 2014)	***—***
miR-79-5p	*—*	*—*	***—***	*—*	*—*	*—*	infected cells (Mayoral et al., 2014)	***—***
miR-8-3p	—	*An. coluzzii* (Bruno et al., 2019)	—	*—*	*Ae. aegypti* (Bryant et al., 2010)	***—***	*Ae.aegypti* cells (Mayoral et al., 2014)	
miR-927-5p	*—*	*—*	***—***	*—*	*—*	***—***	*—*	*—*
miR-927-3p	*—*	*—*	***—***	*—*	*—*	***—***	***—***	***—***
miR-932-5p	*—*	*—*	***—***	*—*	*—*	***—***	*Ae.aegypti* cells (Mayoral et al., 2014)	
miR-956-3p	*—*	*—*	*An. gambiae* (Bryant et al., 2020)	***—***	*—*	*—*	*—*	***—***
miR-957-5p	*—*	*—*	*—*	***—***	*An. gambiae* (Bryant et al., 2019)	*—*	*—*	***—***
miR-965-3p	—	*An. coluzzii* (Bruno et al., 2019)	*—*	*—*	***—***	*—*	*—*	***—***
miR-970-3p	*—*	*—*	***—***	*—*	*—*	*—*	infected cells (Mayoral et al., 2014)	infected cells (Mayoral et al., 2014)
miR-980-3p	*—*	*An. coluzzii* (Bruno et al., 2019)	*—*	*—*	***—***	*—*	*—*	***—***
miR-981-3p	***—***	*An. coluzzii* (Bruno et al., 2019)	*—*	*—*	***—***	*—*	*—*	***—***
miR-989-3p	*An. stephensi*, *Ae. aegypti* (Mead and Tu, 2008), *An. gambiae* (Lampe and Levashina, 2018; Bryant et al., 2019)	**—**	*An. gambiae* (Winter et al., 2007)	*—*	*—*	***—***	*—*	***—***
miR-993-3p	*—*	*—*	*—*	***—***	*An. gambiae* (Bryant et al., 2019)	***—***	***—***	***—***
miR-998-3p	*An. gambiae* (Lampe and Levashina, 2018)	*—*	*—*	***—***	*—*	*—*	*—*	***—***
miR-998-5p	*An. gambiae* (Lampe and Levashina, 2018)	*—*	*—*	***—***	*—*	*—*	*—*	***—***

^&^The term “specific” here indicates that the miRNA is enriched in the corresponding tissue compared with the other tissues from one mosquito species. ^#^The term “specific” here indicates that the miRNA is enriched in either the cytoplasm or nucleus in comparison between the two cell compartments; and for reference (Mayoral et al., 2014), the pathogen used for infection was Wolbachia. “—”, no evidence shows that the miRNA is more abundant in these comparisons.

**Table 6:** Alterations in miRNA abundance in response to infection with different pathogens in various mosquito samples.

miRNA names	Study materials* and changes in expression levels upon pathogen infection						
	CHIKV infection	*Plasmodium* infection	DENV infection	*Wolbachia* infection	ZIKA infection	WNV infection	BTV infection
miR-1-3p	*Ae. albopictus* cells (Shrinet et al., 2014)	***An. anthropophagus* midgut** (Liu et al., 2017), *An. stephensi* (Jain et al., 2014)	*Ae. albopictus* midgut (Su et al., 2017), *Ae. albopictus* (Liu et al., 2015)	***Ae. aegypti* cell cytoplasm** (Mayoral et al., 2014), *Ae. aegypti* cell nucleus (Mayoral et al., 2014)	***Ae. aegypti*** (Saldana et al., 2017)	***—***	***—***
miR-1-5p	***—***	***—***	*—*	*—*	***Ae. aegypti*** (Saldana et al., 2017)	***—***	***—***
miR-10-5p	***Ae.aegypti* saliva, *Ae. albopictus* saliva** (Maharaj et al., 2015)**, *Ae. aegypti*** (Dubey et al., 2017)	***An. anthropophagus* midguts** (Liu et al., 2017), *An. stephensi* (Jain et al., 2014)	*Ae. aegypti* (Liu et al., 2015; Etebari et al., 2015), C6/36 cells (Avila-Bonilla et al., 2017)	*Ae. aegypti* cell nucleus (Mayoral et al., 2014)	***—***	***—***	***—***
miR-100-5p	***Ae. albopictus* cells** (Shrinet et al., 2014), ***Ae. aegypti* saliva**, ***Ae. albopictus* saliva** (Maharaj et al., 2015), *Ae. aegypti* (Saldana et al., 2017)	***An. anthropophagus* midguts** (Liu et al., 2017), *An. stephensi* (Jain et al., 2014)	***Ae. albopictus*** (Yan et al., 2014)	***Ae. aegypti* cell cytoplasm and nucleus** (Mayoral et al., 2014)	***—***	***—***	***—***
miR-1000-5p	***Ae.aegypti* saliva** (Maharaj et al., 2015), *Ae. albopictus* cells (Shrinet et al., 2014)	*An. anthropophagus* midguts (Liu et al., 2017), ***An. stephensi*** (Jain et al., 2014)	***Ae. albopictus* midgut** (Su et al., 2017), *Ae. aegypti* (Campbell et al., 2014)	***Ae. aegypti* cell cytoplasm and nucleus** (Mayoral et al., 2014)	***—***	***—***	***—***
miR-109	*Ae. aegypti* saliva (Maharaj et al., 2015)	*—*	***—***	**—**	***—***	***—***	***—***
miR-11-3p	*Ae. albopictus* cells (Shrinet et al., 2014), *Ae. aegypti* saliva (Maharaj et al., 2015)	***An. anthropophagus* midguts** (Liu et al., 2017), *An. stephensi* (Jain et al., 2014)	***—***	*Ae. aegypti* cell cytoplasm (Mayoral et al., 2014)	***—***	***—***	***—***
miR-11-5p	***Ae. albopictus* cells** (Shrinet et al., 2014)	***—***	***—***	*Ae. aegypti* cell nucleus (Mayoral et al., 2014)	***—***	***—***	***—***
miR-115	*Ae. aegypti* saliva (Maharaj et al., 2015)	***—***	***—***	***—***	***—***	***—***	***—***
miR-117	***Ae. aegypti* saliva** (Maharaj et al., 2015)	***—***	***—***	***—***	***—***	***—***	***—***
miR-1174-3p	*Ae. aegypti* saliva (Maharaj et al., 2015)	***An. stephensi*** (Jain et al., 2014), *An. gambiae* (Winter et al., 2007)	*Ae. albopictus* midgut (Su et al., 2017)	*Ae. aegypti* cell cytoplasm (Mayoral et al., 2014)	***—***	***—***	***—***
miR-1175-5p	*—*	*An. stephensi* (Jain et al., 2014)	***Ae. albopictus* midgut** (Su et al., 2017)	***Ae. aegypti* cell nucleus** (Mayoral et al., 2014), *Ae. aegypti* cell cytoplasm (Mayoral et al., 2014)	***—***	***—***	***—***
miR-1175-3p	*Ae. albopictus* cells (Shrinet et al., 2014), *Ae. aegypti* saliva (Maharaj et al., 2015)	***An. anthropophagus* midguts** (Liu et al., 2017), *An. gambiae* (Winter et al., 2007), *An.stephensi* (Jain et al., 2014)	***Ae. albopictus* midgut** (Su et al., 2017), *Ae. aegypti* (Campbell et al., 2014)	*Ae. aegypti* cell cytoplasm (Mayoral et al., 2014)	***—***	***—***	***—***
miR-11900			*Ae. aegypti* cells (Miesen et al., 2016; Zhang et al., 2017)	*—*	***—***	***—***	***—***
miR-12-5p	***Ae. aegypti* saliva** (Maharaj et al., 2015), *Ae. albopictus* cells (Shrinet et al., 2014)	***An. anthropophagus* midguts** (Liu et al., 2017), *An. stephensi* (Jain et al., 2014)	*Ae. albopictus* midguts (Su et al., 2017)	***Ae. aegypti* cells** (Osei-Amo et al., 2012)	***—***	***—***	***—***
miR-12-3p	***—***	***—***	***—***	***Ae. aegypti* cell nucleus and cytoplasm** (Mayoral et al., 2014)	***—***	***—***	***—***
miR-124-3p	***—***	***An. stephensi*** (Jain et al., 2014)	*Ae. aegypti* (Campbell et al., 2014), C6/36 cells (Avila-Bonilla et al., 2017)	***Ae. aegypti* cell nucleus and cytoplasm** (Mayoral et al., 2014)	***—***	***—***	***—***
miR-1247	***Ae. aegypti* saliva** (Maharaj et al., 2015)	***—***	***—***	***—***	***—***	***—***	***—***
miR-125-5p	***Ae. albopictus* cells** (Shrinet et al., 2014)**, *Ae. aegypti* saliva, *Ae. albopictus* saliva** (Maharaj et al., 2015)	***An. anthropophagus* midguts** (Liu et al., 2017), *An. stephensi* (Jain et al., 2014)	***—***	***Ae. aegypti*** (Hussain et al., 2013)**, *Ae. aegypti* cell cytoplasm and nucleus** (Mayoral et al., 2014)	***—***	***—***	***—***
miR-125-3p	***Ae. albopictus* cells** (Shrinet et al., 2014)	*An. stephensi* (Jain et al., 2014)	***—***	***—***	***—***	***—***	***—***
miR-127	***Ae. albopictus* saliva** (Maharaj et al., 2015)		***—***	***—***	***—***	***—***	***—***
miR-13-3p	***Ae. aegypti* saliva** (Maharaj et al., 2015), C6/36 cells (Shrinet et al., 2014), *Ae. albopictus* saliva (Maharaj et al., 2015)	***An. anthropophagus* midgut** (Liu et al., 2017), *An. stephensi* (Jain et al., 2014)	***—***	***—***	***—***	***—***	*Ae. albopictus* cells (Xing et al., 2016)
miR-13-5p	*Ae. albopictus* cells (Shrinet et al., 2014)	*An. stephensi* (Jain et al., 2014)	***—***	***—***	***—***	***—***	
miR-133-3p	***Ae. aegypti* saliva, *Ae. albopictus* saliva** (Maharaj et al., 2015), *Ae. albopictus* cells (Shrinet et al., 2014)	*An. stephensi* (Jain et al., 2014), *An. anthropophagus* midgut (Liu et al., 2017)	***—***	***—***	***—***	***—***	*Ae. albopictus* cells (Xing et al., 2016)
miR-133-5p		*An. stephensi* (Jain et al., 2014)	***—***	***—***	***—***	***—***	***—***
miR-137-3p	***Ae.albopictus* saliva** (Maharaj et al., 2015), *Ae. albopictus* cells (Shrinet et al., 2014)	***An. stephensi* iBF at 42h** (Jain et al., 2014), ***An. anthropophagus* midguts** (Liu et al., 2017), *An. stephensi* iBF at 5d (Jain et al., 2014)	***—***	***—***	***—***	***—***	***—***
miR-14-3p	***Ae. aegypti* saliva, *Ae. albopictus* saliva** (Maharaj et al., 2015), *Ae. albopictus* cells (Shrinet et al., 2014)	*An. stephensi* (Jain et al., 2014), *An. anthropophagus* midguts (Liu et al., 2017)	***—***	***Ae. aegypti* cell cytoplasm** (Mayoral et al., 2014)	***—***	***—***	***—***
miR-143	***Ae. aegypti* saliva, *Ae. albopictus* saliva** (Maharaj et al., 2015)	*—*	**—**	*—*	**—**	**—**	**—**
miR-15-3p	*—*	*—*	**HEK293 and HeLa cells** (Smith et al., 2017)	*—*	**HEK293 and HeLa cells** (Smith et al., 2017)	**HEK293 and HeLa cells** (Smith et al., 2017)	***—***
miR-157	***Ae. albopictus* saliva** (Maharaj et al., 2015)	*—*	*—*	***—***	***—***	***—***	***—***
miR-1571	***Ae. aegypti* saliva** (Maharaj et al., 2015)	*—*	*—*	***—***	***—***	***—***	***—***
miR-1767		*—*	***Ae. albopictus* midgut** (Su et al., 2017; Su et al., 2019)	***—***	***—***	***—***	***—***
miR-184-3p	***Ae. albopictus* saliva** (Maharaj et al., 2015)**, C6/36 cells** (Dubey et al., 2017), *Ae. aegypti* saliva (Maharaj et al., 2015)	***An. anthropophagus* midguts** (Liu et al., 2017), *An. stephensi* (Jain et al., 2014)	***Ae. albopictus*** (Liu et al., 2015), *Ae. albopictus* midguts (Su et al., 2017)	***—***	***—***	***—***	***—***
miR-1889-3p	***—***	***—***	***Ae. albopictus* and C6/36 cells** (Yan et al., 2014), *Ae. albopictus* (Liu et al., 2015)	***Ae. aegypti* cell cytoplasm** (Mayoral et al., 2014)	***—***	***—***	***—***
miR-1889-5p	***Ae. aegypti* saliva** (Maharaj et al., 2015)	***—***	***—***	*Ae. aegypti* cell cytoplasm (Mayoral et al., 2014)	***—***	***—***	***Ae. albopictus* cells** (Xing et al., 2016)
miR-1890-3p	***Ae. aegypti* saliva** (Maharaj et al., 2015), *Ae. albopictus* cells (Shrinet et al., 2014)	***An. anthropophagus* midguts** (Liu et al., 2017), *An. stephensi* (Jain et al., 2014)	***—***	***Ae. aegypti* cell cytoplasm** (Mayoral et al., 2014)	***—***	***—***	***—***
miR-1891-5p	***Ae. aegypti* saliva**, ***Ae. albopictus* saliva** (Maharaj et al., 2015), *Ae. albopictus* cells (Shrinet et al., 2014)	*An. stephensi* (Jain et al., 2014)	***Ae. albopictus* midgut** (Su et al., 2017), C6/36 cells, *Ae. albopictus* (Yan et al., 2014)	*Ae. aegypti* (Hussain et al., 2011)	***—***	***—***	***—***
miR-190-5p	*Ae. albopictus* cells (Shrinet et al., 2014)	*An. stephensi* (Jain et al., 2014)	**C6/36 cells** (Avila-Bonilla et al., 2017)	*Ae. aegypti* cell cytoplasm (Mayoral et al., 2014)	***—***	***—***	***—***
miR-190-3p	*—*	*An. stephensi* (Jain et al., 2014)	*—*	***—***	***—***	***—***	***—***
miR-193-5p	*—*	*—*	***Ae. albopictus*** (Su et al., 2019)	***—***	***—***	***—***	***—***
miR-193-3p	*Ae. albopictus* cells (Shrinet et al., 2014)	*—*	***—***	***—***	***—***	***—***	***—***
miR-1951		*—*	***Ae. albopictus* midgut** (Su et al., 2019)	***—***	***—***	***—***	***—***
miR-210-3p	***Ae. aegypti* saliva** (Maharaj et al., 2015), *Ae. albopictus* saliva (Maharaj et al., 2015)	*An. stephensi* (Jain et al., 2014), *An. anthropophagus* midguts (Liu et al., 2017)	**C6/36 cells** (Avila-Bonilla et al., 2017)	*Ae. aegypti* (Hussain et al., 2011), C6/36 cells cytoplasm (Mayoral et al., 2014)	***—***	***—***	***—***
miR-210-5p	*Ae. albopictus* cells (Shrinet et al., 2014)	***—***	*Ae. aegypti* (Campbell et al., 2014)	***—***	***—***	***—***	***—***
miR-214	***Ae.aegypti* saliva** (Maharaj et al., 2015), *Ae. albopictus* saliva (Maharaj et al., 2015)	***—***	***—***	***—***	***—***	***—***	***—***
miR-219-5p	*Ae. albopictus* cells (Shrinet et al., 2014)	***—***	***—***	***Ae. aegypti* cell cytoplasm** (Mayoral et al., 2014)	***—***	***—***	***—***
miR-229	***Ae. aegypti* saliva** (Maharaj et al., 2015)	***—***	***—***	***—***	***—***	***—***	***—***
miR-23	***Ae. aegypti* saliva** (Maharaj et al., 2015)	***—***	***—***	***—***	***—***	***—***	***—***
miR-2308	***Ae. albopictus* saliva** (Maharaj et al., 2015)	***—***	***—***	***—***	***—***	***—***	***—***
miR-242	***Ae. aegypti* saliva** (Maharaj et al., 2015)	***—***	***—***	***—***	***—***	***—***	***—***
miR-249	***Ae. albopictus* saliva, *Ae. aegypti* saliva** (Maharaj et al., 2015)	***—***	***—***	***—***	***—***	***—***	***—***
miR-252-5p	***Ae. aegypti* saliva** (Maharaj et al., 2015), *Ae. albopictus* saliva (Maharaj et al., 2015)	*An. stephensi* (Jain et al., 2014), *An. anthropophagus* midgut (Liu et al., 2017)	**C6/36 cells** (Yan et al., 2014), C6/36 cells (Avila-Bonilla et al., 2017)	***Ae. aegypti*** (Hussain et al., 2011), ***Ae. aegypti* cell cytoplasm** (Mayoral et al., 2014)	***—***	***—***	***—***
miR-252-3p	***—***	***—***	*Ae. albopictus* cells (Shrinet et al., 2014)	***—***	***—***	***—***	***—***
miR-263a-3p	***—***	***—***	C6/36 cells (Avila-Bonilla et al., 2017)	***—***	***—***	***—***	***—***
miR-263a-5p	***—***	***—***	*—*	***—***	*Ae. aegypti* (Saldana et al., 2017)	***—***	***—***
miR-275-3p	***Ae. albopictus* cells** (Shrinet et al., 2014)**, *Ae. aegypti* saliva, *Ae.albopictus* saliva** (Maharaj et al., 2015)	*An. stephensi* (Jain et al., 2014), *An. anthropophagus* midgut (Liu et al., 2017)	*Ae. albopictus* (Liu et al., 2015), C7/10 cells, *Ae. albopictus* midgut (Su et al., 2017)	***—***	***—***	***—***	***—***
miR-275-5p	***Ae. albopictus* cells** (Shrinet et al., 2014)	***—***	***—***	***—***	***—***	***—***	***—***
miR-276-3p	***Ae. aegypti* saliva, *Ae. albopictus* saliva** (Maharaj et al., 2015)	***An. anthropophagus* midguts** (Liu et al., 2017), *An. stephensi* (Jain et al., 2014)	***Ae. albopictus* midgut** (Su et al., 2017), *Ae. aegypti* (Etebari et al., 2015), *Ae. albopictus (* Liu et al., 2015)	***Ae. aegypti* cell cytoplasm** (Mayoral et al., 2014)	***—***	***—***	***—***
miR-276-5p	*—*	*An. stephensi* (Jain et al., 2014)	***Ae. albopictus*** (Liu et al., 2015), ***Ae. albopictus* midguts** (Su et al., 2019), *Ae. aegypti* (Campbell et al., 2014), *Ae. albopictus* midgut (Su et al., 2017)	***—***	***—***	***—***	*Ae. albopictus* cells (Xing et al., 2016)
miR-2765-5p	*Ae. albopictus* cells (Shrinet et al., 2014)	***—***	***—***	***Ae. aegypti* cell nucleus** (Mayoral et al., 2014)	***—***	***—***	***—***
miR-277-3p	***Ae. albopictus* cells** (Shrinet et al., 2014), ***Ae. aegypti* saliva, *Ae. albopictus* saliva** (Maharaj et al., 2015)	***An. anthropophagus* midguts** (Liu et al., 2017), *An. stephensi* (Jain et al., 2014)	*Ae. albopictus* (Liu et al., 2015)	***Ae. aegypti*** (Hussain et al., 2011)**, *Ae. aegypti* cell nucleus** (Mayoral et al., 2014)	***—***	***—***	***—***
miR-278-3p	*Ae. albopictus* cells (Shrinet et al., 2014)	***An. anthropophagus* midguts** (Liu et al., 2017), *An. stephensi* (Jain et al., 2014)	***—***	*Ae. aegypti* cell cytoplasm (Mayoral et al., 2014)	***—***	***—***	*Ae. albopictus* cells (Xing et al., 2016)
miR-278-5p	***Ae. albopictus* cells** (Shrinet et al., 2014), ***Ae. aegypti*** (Dubey et al., 2017)	***—***	***—***	*Ae. aegypti* cell cytoplasm (Mayoral et al., 2014)	***—***	***—***	***—***
miR-279-3p	***Ae.aegypti* saliva** (Maharaj et al., 2015), *Ae. albopictus* cells (Shrinet et al., 2014), *Ae. albopictus* saliva (Maharaj et al., 2015)	***An. anthropophagus* midgut** (Liu et al., 2017), *An. stephensi* (Jain et al., 2014)	***—***	***—***	***—***	***—***	***—***
miR-2796-5p	*Ae. albopictus* cells (Shrinet et al., 2014)	*An. stephensi* (Jain et al., 2014)	***Ae. albopictus*** (Liu et al., 2015)	***—***	***—***	***—***	***—***
miR-2779	*Ae. aegypti* (Dubey et al., 2017)	***—***	***—***	***—***	***—***	***—***	***—***
miR-28	***Ae. albopictus* saliva** (Maharaj et al., 2015)	***—***	***—***	***—***	***—***	***—***	***—***
miR-281-3p	***—***	*An. stephensi* (Jain et al., 2014)	***Ae. albopictus*** (Zhou et al., 2014)	***Ae. aegypti*** (Hussain et al., 2011), ***Ae. aegypti* cell cytoplasm** (Mayoral et al., 2014)	***—***	***—***	***—***
miR-281-5p	***Ae. albopictus* saliva** (Maharaj et al., 2015), *Ae. aegypti* saliva (Maharaj et al., 2015)	*An. stephensi* (Jain et al., 2014)	*Ae. albopictus* (Liu et al., 2015; Su et al., 2017)	*Ae. aegypti* cell nucleus (Mayoral et al., 2014)	*—*	*—*	*—*
miR-282-5p	***—***	***—***	*Ae. albopictus* (Su et al., 2017)	***Ae. aegypti* cell nucleus** (Mayoral et al., 2014)	***—***	***—***	***—***
miR-283-5p	***Ae. albopicts* midgut** (Maharaj et al., 2015), ***Ae. albopictus* cells** (Shrinet et al., 2014)	***An. anthropophagus* midguts** (Liu et al., 2017)	***—***	***—***	***—***	***—***	***—***
miR-285-3p	***Ae. aegypti* saliva** (Maharaj et al., 2015), *Ae. albopic- tus* saliva (Maharaj et al., 2015), *Ae. aegypti* (Dubey et al., 2017)	*An. stephensi* (Jain et al., 2014)	***—***	***Ae. aegypti* cell cytoplasm** (Mayoral et al., 2014)	***—***	***—***	***Ae. albopictus* cells** (Xing et al., 2016)
miR-286b-5p	*—*	*—*	C6/36 cells (Avila-Bonilla et al., 2017)	*—*	***—***	*—*	*—*
miR-286a-3p	*—*	*—*	C6/36 cells (Avila-Bonilla et al., 2017)	*—*	***—***	*—*	*—*
miR-2940-3p	*—*	*—*	*Ae. albopictus* (Liu et al., 2015)	*—*	***Ae. aegypti*** (Saldana et al., 2017)	*—*	*—*
miR-2940-5p	*Ae. albopictus* cells (Shrinet et al., 2014), *Ae. aegypti* saliva (Maharaj et al., 2015)	***An. gambiae*** (Biryukova et al., 2014)	*Ae. albopictus* (Liu et al., 2015)	***Ae. aegypti* and *Aedes aegypti* cells** (Hussain et al., 2011; Zhang et al., 2013; Mayoral et al., 2014; Asad et al., 2018)	***—***	C6/36 cells (Slonchak et al., 2014)	*—*
miR-2941-3p	*Ae. albopictus* cells (Shrinet et al., 2014)	***—***	***Ae. albopictus*** (Su et al., 2019), *Ae. albopictus* (Liu et al., 2015), *Ae. albopictus* midgut (Liu et al., 2016)	***Ae. aegypti*** (Hussain et al., 2011)**, *Aedes aegypti* cell nucleus and cytoplasm** (Mayoral et al., 2014)	*Ae. aegypti* (Saldana et al., 2017)	***—***	***—***
miR-2943-5p	***—***	***—***	***Ae. albopictus* midgut** (Liu et al., 2016)	***Ae. aegypti*** (Hussain et al., 2011)	***—***	***—***	***—***
miR-2944a-5p	***Ae. albopictus* cells** (Shrinet et al., 2014)	***An. stephensi*** (Shrinet et al., 2014)	***—***	***—***	***—***	***—***	***—***
miR-2945-3p	***Ae. aegypti* saliva** (Maharaj et al., 2015), *Ae. albopictus* cells (Shrinet et al., 2014)	***An. stephensi*** (Jain et al., 2014)	**C6/36 cells** (Yan et al., 2014)**, *Ae. albopictus*** (Liu et al., 2015), *Ae. aegypti* (Campbell et al., 2014)	*Ae. aegypti* (Hussain et al., 2011), *Ae. aegypti* cells (Mayoral et al., 2014)	***—***	***—***	***—***
miR-2946-3p	*Ae. aegypti* saliva (Maharaj et al., 2015)	***—***	**—**	***Ae. aegypti* cells** (Mayoral et al., 2014)	*Ae. aegypti* (Saldana et al., 2017)	***—***	***Ae. albopictus* cells** (Xing et al., 2016)
miR-2951-5p	*Ae. aegypti* (Dubey et al., 2017)	***—***	***—***	***—***	***—***	***—***	***—***
miR-2a-3p	*—*	***—***	**C6/36 cells** (Avila-Bonilla et al., 2017)	***—***	***—***	***—***	***—***
miR-3	***Ae. aegypti* saliva** (Maharaj et al., 2015)	***—***	***—***	***—***	***—***	***—***	***—***
miR-305-5p	***Ae. aegypti* saliva** (Maharaj et al., 2015), *Ae. albopictus* cell (Shrinet et al., 2014), *Ae.albopictus* saliva (Maharaj et al., 2015)	***An. gambiae* midguts** (Dennison et al., 2015)**, *An. anthropophagus* midguts** (Liu et al., 2017), *An. stephensi* (Jain et al., 2014)	*Ae. aegypti* (Campbell et al., 2014)	*Ae. aegypti* cells (Mayoral et al., 2014)	***—***	***—***	***—***
miR-305-3p	*—*	*An. stephensi* (Jain et al., 2014)	*—*	*—*	***—***	***—***	***—***
miR-306-5p	***Ae. aegypti* saliva** (Maharaj et al., 2015), *Ae. albopictus* cells (Shrinet et al., 2014)	*An. stephensi* (Jain et al., 2014), *An. anthropophagus* midguts (Liu et al., 2017)	*Ae. albopictus* (Liu et al., 2015)	***Ae. aegypti* cell cytoplasm** (Mayoral et al., 2014), *Ae. aegypti* cell nucleus (Mayoral et al., 2014)	*Ae. aegypti* (Saldana et al., 2017)	***—***	***—***
miR-3069	*Ae. aegypti* saliva (Maharaj et al., 2015)	*—*	*—*	**—**	***—***	***—***	***—***
miR-307-3p	***Ae. aegypti* saliva** (Maharaj et al., 2015)	*—*	*—*	**—**	***—***	***—***	***—***
miR-308-3p	***—***	***An. anthropophagus* midguts** (Liu et al., 2017), *An. stephensi* (Jain et al., 2014)	*Ae. aegypti* (Campbell et al., 2014)	*—*	*Ae. aegypti* (Saldana et al., 2017)	***—***	*Ae. albopictus* cells (Xing et al., 2016)
miR-308-5p	**C6/36 cells** (Shrinet et al., 2014)**, *Ae. aegypti* saliva** (Maharaj et al., 2015)	***—***	*—*	***Ae. aegypti*** (Hussain et al., 2011)**, *Ae. aegypti* cell nucleus** (Mayoral et al., 2014)	***Ae. aegypti*** (Saldana et al., 2017)	***—***	***—***
miR-309a-3p	***Ae. aegypti* saliva, *Ae. albopictus* saliva** (Maharaj et al., 2015)	***An. anthropophagus* midgut** (Liu et al., 2017), *An. stephensi* (Jain et al., 2014)	***—***	***—***	***—***	***—***	***—***
miR-31-5p	***Ae. aegypti* saliva** (Maharaj et al., 2015)	***An. anthropophagus* midguts** (Liu et al., 2017)	***—***	***—***	***—***	***—***	***—***
miR-315-5p	***Ae. aegypti* saliva, *Ae. albopictus* saliva** (Maharaj et al., 2015)	*An. stephensi* (Jain et al., 2014)	***—***	***—***	***—***	***—***	***—***
miR-315-3p		*An. anthropophagus* midguts (Liu et al., 2017)	***—***	***—***	***—***	***—***	***—***
miR-317-3p	***Ae. aegypti* saliva** (Maharaj et al., 2015), ***Ae. aegypti*** (Dubey et al., 2017), *Ae. albopictus* saliva (Maharaj et al., 2015)	***An. gambiae (*** Biryukova et al., 2014), *An. stephensi (* Jain et al., 2014), *An. anthropophagus* midguts (Liu et al., 2017)	*Ae. albopictus* (Liu et al., 2015), *Ae. albopictus* midgut (Su et al., 2017)	***Ae. aegypti*** (Hussain et al., 2011), *Ae. aegypti* cell cytoplasm (Mayoral et al., 2014)	***—***	***—***	***—***
miR-317-5p	***Ae. albopictus* cells** (Shrinet et al., 2014)	***—***	***—***	***—***	***—***	***—***	***—***
miR-320	***Ae. aegypti* saliva** (Maharaj et al., 2015)	***—***	**—**	**—**	***—***	**—**	**—**
miR-33-5p	***Ae. aegypti* saliva** (Maharaj et al., 2015), *Ae. albopictus* cell (Shrinet et al., 2014)	***—***	***Ae. albopictus*** (Yan et al., 2014)	***Ae. aegypti* cell cytoplasm** (Mayoral et al., 2014)	**—**	**HEK293 cells** (Slonchak et al., 2015)	**—**
miR-3368-5p	*—*	***—***	*Ae. aegypti* (Campbell et al., 2014)	*—*	***—***	***—***	***—***
miR-34-3p	***Ae. aegypti* cells** (Shrinet et al., 2014)	*—*	***—***	***Ae. aegypti* cell cytoplasm** (Mayoral et al., 2014), *Ae. aegypti* cell nucleus (Mayoral et al., 2014)	***—***	***—***	***—***
miR-34-5p	***Ae. aegypti* saliva** (Maharaj et al., 2015), ***Ae. aegypti*** (Dubey et al., 2017), *Ae. aegypti* cells (Shrinet et al., 2014), *Ae. albopictus* saliva (Maharaj et al., 2015)	***An. anthropophagus* midguts** (Liu et al., 2017), *An. gambiae* (Winter et al., 2007), *An. stephensi* (Jain et al., 2014)	***Ae. aegypti*** (Campbell et al., 2014), ***Ae. albopictus* midgut** (Su et al., 2017), ***Ae. albopictus*** (Su et al., 2019)	***Ae. aegypti*** (Hussain et al., 2011), *Ae. aegypti* cell nucleus (Mayoral et al., 2014)	***—***	***—***	*Ae. albopictus* cells (Xing et al., 2016)
miR-341	***Ae. aegypti* saliva** (Maharaj et al., 2015)	*—*	***—***	***—***	***—***	***—***	***—***
miR-3722-5p	*—*	***—***	*Ae. aegypti* (Campbell et al., 2014)	***—***	***—***	***—***	***—***
miR-375-3p	*Ae. albopictus* cells (Shrinet et al., 2014), *Ae. aegypti* saliva (Maharaj et al., 2015)	***An. anthropophagus* midguts** (Liu et al., 2017), *An. stephensi* (Jain et al., 2014)	*Ae. albopictus* midgut (Su et al., 2017)	***—***	*Ae. aegypti* (Saldana et al., 2017)	***—***	***—***
miR-359	***Ae. aegypti* saliva** (Maharaj et al., 2015)	***—***	***—***	***—***	***—***	***—***	***—***
miR-360	***Ae. aegypti* saliva** (Maharaj et al., 2015)	***—***	***—***	***—***	***—***	***—***	***—***
miR-40	***Ae. aegypti* saliva** (Maharaj et al., 2015)	***—***	***—***	***—***	***—***	***—***	***—***
miR-402	***Ae. aegypti* saliva** (Maharaj et al., 2015)	***—***	***—***	***—***	***—***	***—***	***—***
miR-408	***Ae. aegypti* saliva** (Maharaj et al., 2015)	***—***	***—***	***—***	***—***	***—***	***—***
miR-4110-5p	***—***	***—***	***Ae. albopictus* midgut** (Su et al., 2019)	***—***	***—***	***—***	***—***
miR-424-3p	***—***	***—***	***Ae. albopictus* midgut** (Su et al., 2019)	***—***	***—***	***—***	***—***
miR-4275-5p	***—***	***—***	*Ae. aegypti* (Campbell et al., 2014)	***—***	***—***	***—***	***—***
miR-4448	***—***	***—***	*Ae. albopictus* midgut (Su et al., 2017), *Ae. albopictus* midgut (Su et al., 2019)	***—***	***—***	***—***	***—***
miR-446	***Ae. aegypti* saliva** (Maharaj et al., 2015)	***—***		***—***	***—***	***—***	***—***
miR-4682	***Ae. aegypti* saliva** (Maharaj et al., 2015)	***—***		***—***	***—***	***—***	***—***
miR-47	***Ae. aegypti* saliva** (Maharaj et al., 2015)	***—***		***—***	***—***	***—***	***—***
miR-4728-5p		***—***	***Ae. albopictus* midgut** (Su et al., 2017)	***—***	***—***	***—***	***—***
miR-5	***Ae. aegypti* saliva, *Ae. albopictus* saliva** (Maharaj et al., 2015)	***—***		***—***	***—***	***—***	***—***
miR-5108-5p		***—***	*Ae. aegypti* (Campbell et al., 2014)	***—***	***—***	***—***	***—***
miR-5119-5p		***—***	***Ae. aegypti*** (Campbell et al., 2014)	***—***	***—***	***—***	***—***
miR-5706		***—***	***Ae. albopictus* midgut** (Su et al., 2019)	***—***	***—***	***—***	***—***
miR-576	***Ae. aegypti* saliva** (Maharaj et al., 2015)	***—***	***—***	***—***	***—***	***—***	***—***
miR-6	***Ae. aegypti* saliva** (Maharaj et al., 2015)	***—***	***—***	***—***	***—***	***—***	***—***
miR-62	*Ae. albopictus* saliva (Maharaj et al., 2015)	***—***	***—***	***—***	***—***	***—***	***—***
miR-620	***Ae. aegypti* saliva** (Maharaj et al., 2015)	***—***	***—***	***—***	***—***	***—***	***—***
miR-622		***—***	***Ae. albopictus* midgut** (Su et al., 2017; Su et al., 2019)	***—***	***—***	***—***	***—***
miR-69	***Ae. aegypti* saliva** (Maharaj et al., 2015)	***—***	***—***	***—***	***—***	***—***	***—***
miR-7-5p	C6/36 cells (Shrinet et al., 2014)	*An. stephensi* (Jain et al., 2014)	***—***	***Ae. aegypti* cell nucleus** (Mayoral et al., 2014)	***—***	***—***	*Ae. albopictus* cells (Xing et al., 2016)
miR-71-5p	***Ae. aegypti*** (Dubey et al., 2017)	*An. stephensi* (Jain et al., 2014)	***—***		***Ae. aegypti*** (Saldana et al., 2017)	***—***	***—***
miR-71-3p	***Ae. aegypti* saliva** (Maharaj et al., 2015), C6/36 cells (Shrinet et al., 2014), *Ae. albopictus* saliva (Maharaj et al., 2015)	*An. stephensi* (Jain et al., 2014), *An. anthropophagus* midguts (Liu et al., 2017)	*Ae. albopictus* midgut (Su et al., 2017)	***Ae. aegypti* cell cytoplasm** (Mayoral et al., 2014)	***—***	***—***	***—***
miR-778	***Ae. aegypti* and *Ae. albopictus* midgut** (Maharaj et al., 2015)	***—***	***—***	***—***	***—***	***—***	***—***
miR-79-5p	***—***	***—***	***—***	***Ae. aegypti* cell cytoplasm** (Mayoral et al., 2014)	***—***	***—***	***—***
miR-8-3p	***Ae. albopictus* saliva** (Maharaj et al., 2015), *Ae. albopictus* cells (Shrinet et al., 2014)	*An. stephensi* (Jain et al., 2015; Guo et al., 2017)	*Ae. albopictus* (Liu et al., 2015), C7/10 cells, *Ae. albopictus* midgut (Su et al., 2017)	***Ae. aegypti*** (Hussain et al., 2011), *Ae. aegypti* cell cytoplasm (Mayoral et al., 2014)	***—***	***—***	***—***
miR-8-5p	***Ae. albopictus* cells** (Shrinet et al., 2014)	*An. stephensi* (Jain et al., 2014)	*Ae. albopictus* (Liu et al., 2015)	*Ae. aegypti* cell cytoplasm (Mayoral et al., 2014)	***—***	***—***	***—***
miR-80	*Ae.aegypti* saliva (Maharaj et al., 2015)	***—***	***—***	***—***	***—***	***—***	***—***
miR-87-3p	***Ae. aegypti* saliva** (Maharaj et al., 2015), *Ae. albopictus* cells (Shrinet et al., 2014)	*An. anthropophagus* midgut (Liu et al., 2017)	***Ae. albopictus* midgut** (Su et al., 2017), **C6/36 cells** (Avila-Bonilla et al., 2017)	***—***	***—***	***—***	***—***
miR-87-5p	***—***	***—***	*Ae. albopictus* midgut (Su et al., 2019)	***—***	***—***	*—*	*—*
miR-927-5p	***Ae. albopictus* cell** (Shrinet et al., 2014), ***Ae. aegypti* midgut, *Ae. albopictus* midgut** (Maharaj et al., 2015)	*An. stephensi* (Jain et al., 2014)	*Ae. albopictus* (Liu et al., 2015), **C6/36 cells** (Avila-Bonilla et al., 2017; Avila-Bonilla et al., 2020)	***—***	***—***	*—*	*—*
miR-927-3p	***—***	*An. stephensi* (Jain et al., 2014)	***Ae. albopictus* midgut** (Su et al., 2017)	***—***	***—***	***—***	***Ae. albopictus* cells** (Xing et al., 2016)
miR-929-5p	***—***	*An. stephensi* (Jain et al., 2014)	***—***	***—***	***—***	*—*	*—*
miR-92b-3p	***—***	***—***	C6/36 cells (Avila-Bonilla et al., 2017)	***—***	***—***	***—***	***—***
miR-92a-3p	***—***	***An. gambiae*** (Dennison et al., 2015)	***—***	***—***	***—***	***Cx.Quinquefa- sciatus*** (Skalsky et al., 2010)	***—***
miR-932-3p	***—***		***—***	**mosquito cell nucleus** (Mayoral et al., 2014)	***—***	**—**	***—***
miR-932-5p	***Ae. aegypti* saliva** (Maharaj et al., 2015), C6/36 cells (Shrinet et al., 2014), *Ae. albopictus* saliva (Maharaj et al., 2015)	***An. stephensi*** (Jain et al., 2014)	*Ae. aegypti* (Campbell et al., 2014)	*Ae. aegypti* cell cytoplasm (Mayoral et al., 2014)	***—***	*—*	***—***
miR-956-3p	***—***	***—***	*Ae. albopictus* midgut (Su et al., 2017)	*—*	*—*	*—*	*—*
miR-957-3p	***Ae. aegypti* saliva**, ***Ae. albopictus* saliva** (Maharaj et al., 2015)	*An. stephensi* (Jain et al., 2014), *An. anthropophagus* midgut (Liu et al., 2017)	*Ae. albopictus* (Liu et al., 2015)	*—*	*—*	*Cx.Quinquefa- sciatus* (Skalsky et al., 2010)	*—*
miR-965-3p	—	—	*—*	***Ae. aegypti* cell nucleus and cytoplasm** (Mayoral et al., 2014)	***—***	***—***	***—***
miR-970-3p	***Ae. aegypti* saliva** (Maharaj et al., 2015)	*An. stephensi* (Jain et al., 2014), *An. anthropophagus* midguts (Liu et al., 2017)	**C6/36 cells** (Avila-Bonilla et al., 2017)	***—***	—	—	—
miR-976-5p			***Ae. albopictus* midgut** (Su et al., 2019)	*—*	***—***	***—***	***—***
miR-980-3p	***Ae.aegypti* saliva** (Maharaj et al., 2015), *Ae. albopictus* cells (Shrinet et al., 2014)	*An. stephensi* (Jain et al., 2014), *An. anthropophagus* midgut (Liu et al., 2017)	*—*	*—*	*Ae. aegypti* (Saldana et al., 2017)	***Cx.quinquefa- sciatus*** (Skalsky et al., 2010)	*Ae. albopictus* cells (Xing et al., 2016)
miR-980-5p	***Ae. albopictus* cells** (Shrinet et al., 2014)	*—*	***—***	***—***	*—*	*—*	*—*
miR-981-3p	***—***	***An. anthropophagus* midguts** (Liu et al., 2017), *An. stephensi* (Jain et al., 2014)	*—*	—	***—***	***—***	***—***
miR-988-5p	*Ae. albopictus* cells (Shrinet et al., 2014)		*Ae. albopictus* midgut (Su et al., 2017)	***—***	*—*	*—*	*—*
miR-988-3p	***Ae. albopictus* cells** (Shrinet et al., 2014)	*An. stephensi* (Jain et al., 2014)	*—*	*Ae. aegypti* (Hussain et al., 2011)	*—*	*—*	*—*
miR-989-3p	*Ae. aegypti* (Dubey et al., 2017)	***An. gambiae* midgut** (Winter et al., 2007; Dennison et al., 2015), *An. gambiae* leftover (Winter et al., 2007)	***Ae. albopictus* midgut** (Su et al., 2019), *Ae. albopictus* midguts (Liu et al., 2016), *Ae. albopictus* (Su et al., 2017)	*Ae. aegypti* (Hussain et al., 2011), *Ae. aegypti* cell nucleus and cytoplasm (Mayoral et al., 2014)	***Ae. aegypti*** (Saldana et al., 2017)	*Cx.quinquef- asciatus* (Skalsky et al., 2010)	*—*
miR-993-5p	*—*	*An. stephensi* (Jain et al., 2014)	*—*	***—***	***—***	***—***	***—***
miR-993-3p	*—*	*An. stephensi* (Jain et al., 2014)	***Ae. albopictus* midgut** (Liu et al., 2015)	***—***	***—***	***—***	***—***
miR-996-3p	***Ae. albopictus* saliva** (Maharaj et al., 2015), C6/36 cells (Shrinet et al., 2014), *Ae. aegypti* saliva (Maharaj et al., 2015)	*An. stephensi* (Jain et al., 2014)	***—***	***Ae. aegypti* cell cytoplasm** (Mayoral et al., 2014)	***—***	***—***	***—***
miR-996-5p	***—***	***An. anthropophagus* midgut** (Liu et al., 2017)	***—***	***—***	***—***	***—***	***—***
miR-988-5p	***—***	***—***	***—***	***Ae. aegypti* cell nucleus** (Mayoral et al., 2014)	***—***	***—***	***—***
miR-988-3p	***—***	***—***	***—***	***Ae. aegypti* cell cytoplasm** (Mayoral et al., 2014)	***—***	***—***	***—***
miR-998-3p	***Ae. albopictus* cells** (Shrinet et al., 2014), ***Ae. aegypti* saliva** (Maharaj et al., 2015), *Ae. aegypti* (Dubey et al., 2017)	***An. anthropophagus* midguts** (Liu et al., 2017), *An. stephensi* (Jain et al., 2014)	***Ae. albopictus* midgut** (Su et al., 2017)		***—***	***—***	***—***
miR-998-5p	***Ae. albopictus* cells** (Shrinet et al., 2014)	***—***	***—***	***—***	***—***	***—***	***—***
miR-999-3p	*Ae. albopictus* cells (Shrinet et al., 2014), *Ae. aegypti* saliva, *Ae. albopictus* saliva (Maharaj et al., 2015)	*An. stephensi* (Jain et al., 2014)	*Ae. aegypti* (Campbell et al., 2014)	***—***	***—***	***—***	***—***
miR-9a-5p	*—*	*—*	C6/36 cells (Avila-Bonilla et al., 2017)	***—***	***—***	***—***	***—***
miR-iab-4-5p	***—***	***An. anthropophagus* midguts** (Liu et al., 2017)	***—***	***—***	***—***	***—***	***—***
miR-iab-8-5p	*Ae. albopictus* cells (Shrinet et al., 2014)	***—***	***—***	***—***	***—***	***—***	***—***
bantam-3p	***Ae. aegypti* saliva** (Maharaj et al., 2015), C6/36 cells (Shrinet et al., 2014), *Ae. albopictus* saliva (Maharaj et al., 2015)	***An. anthropophagus* midguts** (Liu et al., 2017), *An. stephensi* (Jain et al., 2014)	*Ae. aegypti* (Campbell et al., 2014)	***Ae. aegypti* cell nucleus** (Mayoral et al., 2014)	***—***	***—***	***—***
bantam-5p	*—*	*An. stephensi* (Jain et al., 2014)	*Ae. aegypti* (Etebari et al., 2015), *Ae. albopictus* (Liu et al., 2015)	***Ae. aegypti* cell nucleus** (Mayoral et al., 2014), *Ae. aegypti* cell cytoplasm (Mayoral et al., 2014)	***—***	***—***	***—***
let-7-5p	*Ae. albopictus* cells (Shrinet et al., 2014), *Ae. albopictus* saliva (Maharaj et al., 2015)	*An. stephensi* (Jain et al., 2014), *An. anthropophagus* midguts (Liu et al., 2017)	***Ae. albopictus*, C6/36 cells** (Yan et al., 2014), *Ae. aegypti* (Campbell et al., 2014), *Ae. albopictus* (Liu et al., 2015; Su et al., 2017)	***Ae. aegypti* cells** (Hussain et al., 2011)**, nucleus, cytoplasm of cells** (Mayoral et al., 2014)	***—***	***—***	***—***

*The “study materials” are written in two styles, namely **bold** and nonbold, which means that the miRNAs are upregulated or downregulated upon infection with the corresponding microbes or pathogens infection, respectively. “—”, no related data on upregulation or downregulation is available.

## Steps of the Research Roadmap

**Figure 1:**
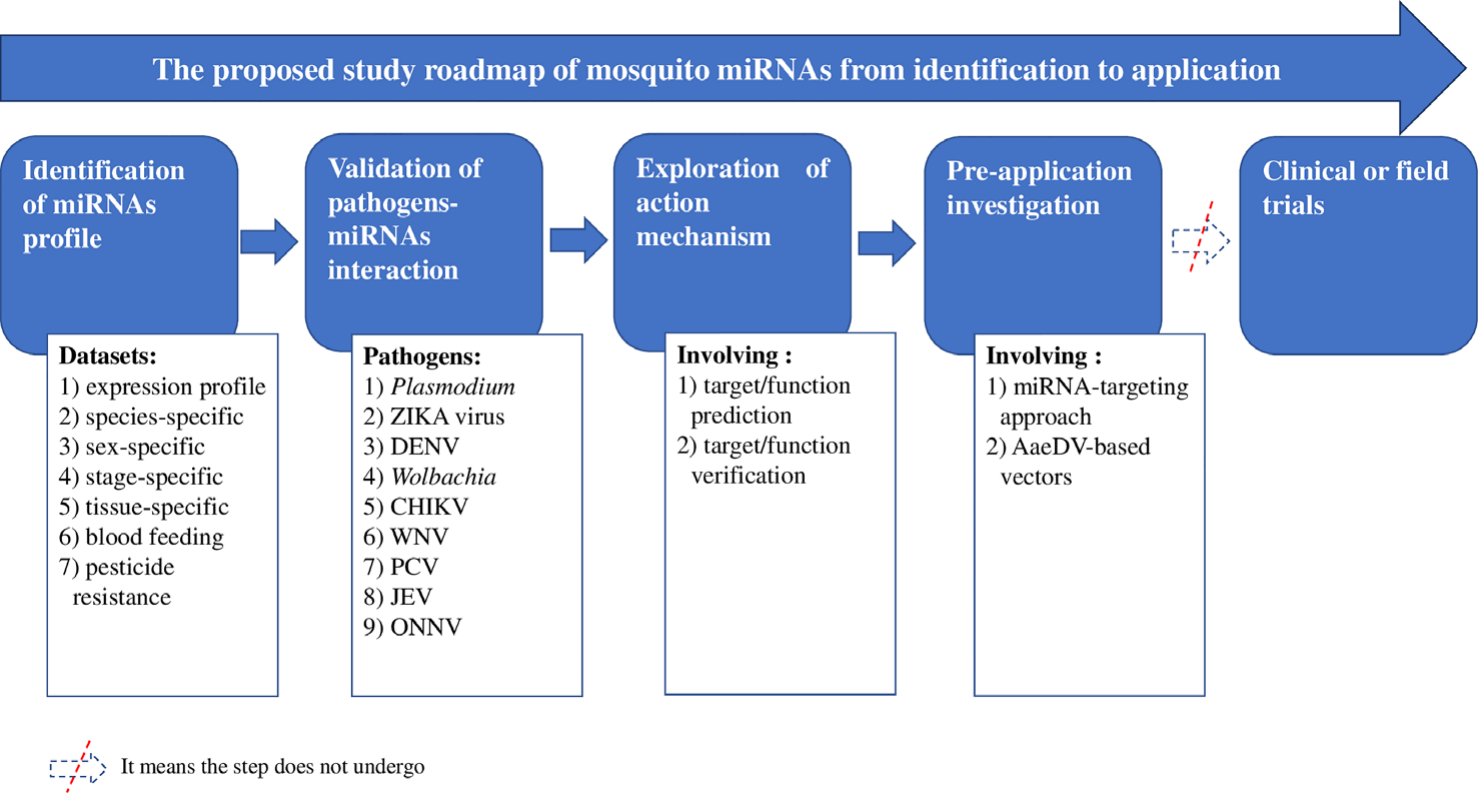
Overall advances in studies of mosquito miRNAs. ZIKA, Zika virus; DENV, Dengue virus; CHIKV, Chikungunya virus; WNV, West Nile virus; ONNV, o’nyong’nyong virus; JEV, Japanese encephalitis virus; PCV, Palm Creek virus; AaeDVs, *Ae. aegypti* densoviruses.

Progress in studying individual miRNAs with annotated names in the database was tracked (**Tables 1** **–** **7** and **S1**), and an overview of study advances is provided in **Figure 1**. The exploration of miRNA-based approaches proceeded through the following four steps along the proposed research roadmap: identifying mosquito miRNAs (**Tables 1**–**5**); validating pathogen- miRNA interactions (**Tables 6** and **S1**); exploring the mechanism of action, which refers mainly to target prediction and verification (**Tables 7** and **S1**); and performing preapplication investigations (Liu P. et al., 2016). These steps involved the 20 items listed in **Figure 1**, for example, interactions between miRNAs and *Plasmodium*, dengue virus (DENV), Zika virus (ZIKA), Chikungunya virus (CHIKV), *Wolbachia*, West Nile virus (WNV), Palm Creek virus (PCV), Japanese Encephalitis virus (JEV), and o’nyong’nyong virus (ONNV).

**Table 7:** miRNAs with validated targets that exert promotive or inhibitory effects on pathogens.

miRNA names	Validated targets	Validated promotive or inhibitory effect on pathogens
miR-12-5p	MCM6 and MCT1 (Osei-Amo et al., 2012)	Facilitating the *Wolbachia* infection (Osei-Amo et al., 2012; Maharaj et al., 2015), enhancing CHIKV infection (Maharaj et al., 2015)
miR-125-5p	—	Enhancing CHIKV infection (Maharaj et al., 2015)
miR-2944a-5p	vps-13 and CHIKV (Dubey et al., 2019)	Repressing CHIKV replication (Dubey et al., 2019)
miR-184-3p	AAEL002512, AAEL005741 (Zhang et al., 2017)	Inhibiting CHIKV infection (Maharaj et al., 2015)
miR-375-3p	Cactus, kinesin, prohibitin, DEAD box ATP-depen-dent RNA helicase, REL1, hypothetical protein (Hussain et al., 2013)	Enhancing DENV-2 infection (Hussain et al., 2013), repressing CHIKV replication (Maharaj et al., 2015).
miR-1767	—	Enhancing DENV-2 replication (Su et al., 2019)
miR-252-5p	DENV E protein gene (Yan et al., 2014)	Inhibiting DENV replication (Yan et al., 2014)
miR-281-3p	5’-UTR of DENV-2 (Zhou et al., 2014)	Enhancing DENV-2 replication (Zhou et al., 2014)
miR-4448	—	Inhibiting DENV-2 infection (Su et al., 2019)
miR-4728-5p	—	Enhancing DENV infections (Su et al., 2017)
miR-927-5p	FLN (Avila-Bonilla et al., 2020)	Reguating antimicrobial peptides, promoting DENV infection (Avila-Bonilla et al., 2020)
miR-2940-5p	AaDnmt 2 (Zhang et al., 2013), metalloprotease m41 FtsH gene (Slonchak et al., 2014), AaArgM3 (Zhang et al., 2014)	Facilitating *Wolbachia* infection (Hussain et al., 2011; Zhang et al., 2014) and subsequent inhibition of DENV replication (Zhang et al., 2013), restricting WNV replication (Slonchak et al., 2014)
miR-276-5p	Branched-chain amino acid transferase (Lampe et al., 2019)	Enhancing DENV-2 infection (Su et al., 2019), prolonging AA catabolism, and then inhibiting development of sporozoites (Lampe et al., 2019)
miR-137-3p	—	Inhibiting *Plasmodium* infection (Jain et al., 2014)
miR-14-3p	3’UTR-binding sites of GCE mRNA (Qu et al., 2017)	*P. falciparum* and gut microbiota agonist (Dong et al., 2020)
miR-305-5p	—	*P. falciparum* and gut microbiota agonist (Dennison et al., 2015; Dong et al., 2020)
miR-124-3p	Dynamin 2 (Yang et al., 2016), PGRP-LD (Feng et al., 2018b)	Inhibiting JEV infection (Yang et al., 2016)
miR-34-5p	Activation of type I interferon signaling (Smith et al., 2017)	Role in vector competence (Winter et al., 2007), inhibits multiple flaviviruses (Smith et al., 2017)

MCT1, monocarboxylate transporter; MCM6, DNA replication licensing; UTR, untranslated region; FLN, cytoskeleton; AaArgM3, protein arginine methyltransferase 3.

However, no clinical or field trial has been reported, indicating that the miRNA-based approach may have encountered a bottleneck of application in mosquito-borne disease prevention and control, although several attempts to establish application models have been conducted (Heiss et al., 2011; Tsetsarkin et al., 2015; Tsetsarkin et al., 2016a; Tsetsarkin et al., 2016b). The details of significant progress achieved at each step are reviewed below.

## Advances at Each Step of the Research Roadmap

### Identification of miRNA Profiles in Mosquitoes

The first step in the research roadmap is to understand mosquito miRNA profiles. Currently, studies of mosquito miRNA profiles focus mainly on mosquito miRNA identification (Mead and Tu, 2008; Skalsky et al., 2010; Gu et al., 2013; Jain et al., 2014; Castellano et al., 2015; Etebari et al., 2015; Hu et al., 2015; Allam et al., 2016; Su et al., 2017; Carissimo et al., 2018; Feng et al., 2018a) with the detection of spatial and temporal expression patterns (Jain et al., 2014; Yen et al., 2018), the functional arm between the 5’ and 3’ ends (Skalsky et al., 2010; Biryukova et al., 2014; Etebari et al., 2015), the production of miRNAs with varying lengths and sequences (i.e., isomiRs) (Skalsky et al., 2010; Biryukova et al., 2014; Castellano et al., 2015; Nouzova et al., 2018), and miRNA clusters (Biryukova et al., 2014). At this step in the roadmap, variations in the spatial and temporal expression of miRNAs have been observed by analyzing several factors. (i) Mosquito species: For example, miR-282-5p was found to be conserved in *Ae. aegypti* and *An. gambiae* but not in *Cx. quinquefasciatus* (Gu et al., 2013) (**Table 2**). Additionally, miR-1175-3p expression has been found to display opposite trends (upregulated or downregulated by blood feeding) in *Aedes albopictus* and *Anopheles gambiae*, and the corresponding molecular mechanisms may also be different in each species (Winter et al., 2007; Su et al., 2017) (**Table 4**). (ii) Sexes: For example, miR-989-5p was found to be restricted in female *Anopheles coluzzii* (Bruno et al., 2019) (**Table 3**). (iii) Developmental stages: Notably, miR-1-3p was found to be enriched in the pupa of female *Anopheles stephensi* (Jain et al., 2015) compared to the larvae and adult (**Table 4**). (iv) Blood feeding and insecticide resistance statuses: The expression of miR-999-3p was found to be downregulated in deltamethrin-resistant *Culex pipiens* (Hong et al., 2014) (**Table 4**). (v) Tissues: For example, miR-998-5p was found to be specifically expressed in the ovary of *An. gambiae* (Lampe and Levashina, 2018) (**Table 5**), and miR-8-3p was found to be particularly enriched in the salivary glands in *An. coluzzii* (Bruno et al., 2019) but in the fat body in *Aedes aegypti* (Bryant et al., 2010) (**Table 5**). Moreover, in addition to these specificities, miRNAs may even exhibit cellular cytoplasm- or nucleus- specificity (Mayoral et al., 2014) (**Table 5**).

More examples based on individual miRNAs are noted in **Tables 2** **–** **5**. Overall, the expression levels of miRNAs are regulated by complicated factors, including mosquito species, sexes, developmental stages, tissues or organs, aging, blood feeding, and so on (**Tables 2** **–** **5**). In addition to differences in expression levels, the preferred or functional arm also varies among these factors in terms of the change in 5p/3p ratio or even dominant arm shifts (Skalsky et al., 2010; Biryukova et al., 2014; Castellano et al., 2015). For example, the 5p/3p ratios of miR-956-3p and miR-219-5p are significantly reduced by blood feeding (Biryukova et al., 2014). Moreover, isomiR production based on acylation, uridylation, adenine and uracil extension/addition can be induced by blood feeding and insecticide resistance (Skalsky et al., 2010; Biryukova et al., 2014).

### miRNA-Pathogen Interactions in the Mosquitoes

The second step in the research roadmap always begins with an observation of statistical correlations between miRNA regulation and pathogen infection in mosquitoes. The pathogens primarily include *Plasmodium*, DENV, CHIKV, *Wolbachia*, Zika virus, WNV, JEV, PCV and ONNV (**Table 6**). The miRNA abundance may vary upon pathogen infection in mosquitoes according to differences in the studied material, e.g., miR-10-5p is upregulated in CHIKV-infected *Ae. aegypti* (Dubey et al., 2017); conversely, it is downregulated in DENV-infected *Ae. aegypti* (Liu et al., 2015; Etebari et al., 2015) (**Table 6**). More importantly, a few miRNAs were found to exhibit similar regulation patterns in different independent studies, and the repeatability of these results makes them more reliable (**Table 6**), as described in the following examples below. The miR-10-5p, -125-5p, -143, -275-3p, -277-3p, -308-5p, and -927-5p have been consistently shown to be upregulated upon CHIKV infection (**Table 6**). Downregulation of miR-133-3p, -14-3p, -252-5p, -275-3p, miR-306-3p, -71-3p, -957-3p, -970-3p, -980-3p, or let-7-5p has been observed upon *Plasmodium* infection (**Table 6**). Upregulation of miR-1767, -34-5p, or -622 and downregulation of miR-1-3p, -275-3p, -317-3p, -4448, -8-3p, or bantam-5p have been detected upon DENV infection. Upregulation of miR-125-5p, -252-5p, -277-3p, -281-3p, -2940-5p, -2941-3p, -308-5p, or let-7-5p and downregulation of miR-210-3p, -2945-3p, or -989-3p have been observed upon *Wolbachia* infection. Notably, miR-2940-5p, -375-3p, -87-3p, -988-5p, and -999-3p are consistently regulated by CHIKV and DENV, which may provide insight into coregulation by these two pathogens and the subsequent codevelopment of miRNA-based approaches for transmission control. More specifically, miR-2940-5p is inversely regulated by DENV and *Wolbachia*, consistent with the results that *Wolbachia* uses miRNA-2940-5p to inhibit DENV infection in *Ae. aegypti* (Hussain et al., 2011; Zhang et al., 2013) (**Table 6**).

As in the first study step in the roadmap, in addition to differences in expression levels, changes in 5p/3p ratio, dominant arm shifts, and isomiR production can be modified by pathogen infection (Etebari et al., 2015).

JEV, PCV and ONNV pathogens do not appear in the summary presented in **Table 6**. Except for a study showing that miR-124 inhibits JEV replication in PK15 porcine kidney epithelial cells (Yang et al., 2016), no report that has indicated that miRNAs are statistically correlated with JEV infection in mosquito or mosquito cells. PCV and ONNV infection exert remarkably limited effects on the mosquito miRNA profile; therefore, miRNAs may not play an important role in the interaction of PCV with *Ae. aegypti* (Lee et al., 2017) or ONNV with *Anopheles coluzzii* (Carissimo et al., 2018). Thus, researchers are currently unable to select a miRNA as an ideal candidate to establish a miRNA-based approach for the control of three mosquito-borne diseases.

After statistical correlations between miRNA alterations and pathogen infection being observed, their causal relationship should be confirmed (**Tables 7** and **S1**). Overexpression or suppression of a miRNA is the most widely used approach to study causality (Jones-Rhoades et al., 2006). Eighteen miRNAs have been validated to exert promotive or inhibitory effects on CHIKV (Maharaj et al., 2015; Dubey et al., 2019), DENV (Hussain et al., 2013; Zhang et al., 2013; Yan et al., 2014; Zhou et al., 2014; Su et al., 2017; Su et al., 2019; Avila-Bonilla et al., 2020), WNV (Slonchak et al., 2014), *Plasmodium* (Jain et al., 2014; Dennison et al., 2015; Lampe et al., 2019; Dong et al., 2020), *Wolbachia* (Hussain et al., 2011; Zhang et al., 2014), or JEV (Yang et al., 2016) infections *via* these types of experiments (**Table 7**). Notably, miR-2940-5p restricts the replication of both WNV and DENV in mosquitoes (Zhang et al., 2013; Slonchak et al., 2014), and miR-375-3p exerts the opposite effect on DENV-2 and CHIKV (Hussain et al., 2013; Maharaj et al., 2015). It is easy to find that the upregulating and downregulating miRNAs in response to pathogen infection co-exist in the mosquito (**Table 7**). No miRNA has been reported to induce multi-antipathogen effects on the two kinds of flaviviruses and *Plasmodium* protozoans. A more detailed description of the progress achieved by studies examining miRNA-pathogen interactions in mosquitoes is presented in **Table S1**.

### Exploration of the Mechanism of Action

Research on the mechanism of action mostly focuses on the prediction and verification of miRNA targets or functions through genetic disruption methods. Bioinformatic analysis tools, such as TargetScan, PITA and RNAhybrid, are always used for target prediction. The verification methods usually include the transfection of miRNA-specific antagomirs into mosquito cells, miRNA mimic/inhibitor microinjection in mosquitoes, real-time quantitative polymerase chain reaction (qRT-PCR**)**, or luciferase assays (Liu B. et al., 2016; Ma et al., 2017; Nouzova et al., 2018; Yen et al., 2019; Avila-Bonilla et al., 2020; Fu et al., 2020). The most recent method applied to elucidate the targets and biological functions of mosquito miRNAs is high-throughput sequencing of covalent ligation of endogenous Argonaute-bound RNAs isolated by crosslinking and immunoprecipitation (CLEAR-CLIP). In this assay, the miRNA and its target mRNA are joined in the purified RNA-induced silencing complex (RISC) complex to form one chimeric molecule. Analysis of the chimeric miRNA-target molecule among the RNA molecules associated with Argonaute **(**AGOs**)** proteins facilitates the systematic identification of miRNA-target interactions (Dong et al., 2020).

The miRNAs noted in bold in **Table S1** have been confirmed to contribute to blood digestion (Bryant et al., 2010; Jain et al., 2014), egg development (Bryant et al., 2010; Puthiyakunnon et al., 2013; Jain et al., 2014; Lucas et al., 2015a; Zhang et al., 2017), ovary development (Ling et al., 2017), larval eclosion (Puthiyakunnon et al., 2013; Feng et al., 2018b), reproduction (Zhang et al., 2016; Fu et al., 2017), the stability and nuclear translocation of AGO1 (Hussain et al., 2013), lipid accumulation (Batz et al., 2017), metabolism (Ling et al., 2017), host-pathogen interactions (Yan et al., 2014; Lucas et al., 2015b; Dubey et al., 2019; Yen et al., 2019), and insecticide resistance (Hong et al., 2014) (**Table S1**).

In the canonical mechanism of action of miRNAs, mature miRNAs guide the RISC to the 3’ untranslated regions (UTRs) of target mRNAs *via* complementary base pair interactions, thus regulating the expression of target genes (Hammond et al., 2000; Lee et al., 2002; Lee et al., 2003). Most miRNA-target (mRNA) interactions are consistent with the canonical action mechanism; however, exceptions have been identified for miRNA-virus interactions (e.g., DENV and CHIKV) in terms of the target type or regulatory outcome. First, during arbovirus infection, mosquito miRNAs can directly bind to the 3’-UTR of the viral genome (not necessary an mRNA), regulating virus replication (Yan et al., 2014; Lucas et al., 2015b; Dubey et al., 2019; Yen et al., 2019), which differs from the canonical mechanism of action. However, the mechanisms underlying mosquito miRNA-*Plasmodium* interactions are always consistent with the canonical mechanism of action, namely, miRNAs generally bind to mosquito immunity- or development-related mRNAs, indirectly regulating pathogen infection (Jain et al., 2014; Dennison et al., 2015; Dong et al., 2020) (**Table S1**). However, the exact mechanism of translational or viral repression remains unclear (Winter et al., 2007). Second, miRNAs always negatively regulate their targets by inducing mRNA cleavage (Yekta et al., 2004) or degradation (Eichhorn et al., 2014), or by repressing translation (Fabian and Sonenberg, 2012); however, positive regulation by miRNAs is repeatedly observed in mosquitoes (Hussain et al., 2013; Zhou et al., 2014; Maharaj et al., 2015; Su et al., 2019). In addition to repressing gene expression, miRNAs can also induce the expression of genes with complementary promoter sequences, switching these genes from repressed to activated (Hussain et al., 2013; Zhou et al., 2014; Maharaj et al., 2015; Su et al., 2019).

More interestingly, the miRNA-target interaction may involve a complex network. A network was observed among clusters of miR-2-3p, miR-13-3p, miR-71-5p, CYP9J35 (a target of miR-2-3p and -13-3p), and CYP325BG3 (a target of miR-71-5p) in insecticide resistant *Cx. pipiens* (Hong et al., 2014).

Moreover, infection with one pathogen affects coinfection with another pathogen in mosquitoes, especially for *Wolbachia* or engineered mosquito densoviruses (MDVs), which can modify host miRNA profiles or use a specific host miRNA to manipulate pathogen invasion in mosquitoes (Osei-Amo et al., 2012; Maharaj et al., 2015; Liu P. et al., 2016).

### Preapplication Investigation

Of the 1635 putative or mature miRNAs reported in mosquitoes, only a few have advanced to the step of preapplication investigations, and the names of these miRNAs are italicized in **Table S1**. The first attempt to establish an application is to exploit the vector specificity and stability of MDVs, which are restricted to mosquitoes. Anti-miRNA sponges targeting endogenous let-7-5p and miR-210-3p were introduced into MDVs in *Ae. aegypti* (noted as **AaeDV**-based vectors in **Figure 1**), and both sponges downregulated the expression levels of these miRNAs. According to the study, this recombinant vector is useful to purposefully inhibit or promote the expression of endogenous miRNAs and subsequently regulate pathogen infection in mosquitoes (Liu P. et al., 2016). This study is similar to a study research that used a plasmid construction technique to express artificial miRNAs that inhibit JEV (Wu et al., 2011) and DENV (Xie et al., 2013) *in vitro* or impede mosquito reproduction and embryonic development (Biedler et al., 2014).

Given the specificity of miRNA-virus interactions in which mosquito miRNAs can directly inhibit the virus *via* complementary base pair interactions, methods to introduce sequences complementary to mosquito miRNAs (noted as miRNA-targeting approaches in **Figure 1**) into arboviruses have been established (Heiss et al., 2011; Tsetsarkin et al., 2015; Tsetsarkin et al., 2016a; Tsetsarkin et al., 2016b). The introduction of a single copy of a miRNA target sequence into the DENV genome was shown to lead to the reduction of DENV 4 replication *in vivo* and *in vitro* (Heiss et al., 2011; Tsetsarkin et al., 2015), consistent with the results of a similar study with another pathogen, JEV (Yen et al., 2013). More interestingly, multiple insertions of heterologous target sequences of different miRNAs into the virus were shown to increase virus attenuation, whereas the insertion of two or three copies of homologous sequence (the same miRNAs) into the virus did not increase virus attenuation (Tsetsarkin et al., 2016a; Tsetsarkin et al., 2016b).

The two preapplication investigations indicate the possible application of miRNA-based approaches, e.g., 1) expressing a miRNA inhibitor in vector mosquitoes by establishing genetically modified mosquitoes, subsequently reducing the fitness between mosquitoes and pathogens and interrupting the transmission of mosquito-borne pathogens (Heiss et al., 2011; Tsetsarkin et al., 2015; Tsetsarkin et al., 2016a; Tsetsarkin et al., 2016b); and 2) inserting specific miRNA target sequences into the flavivirus genome, resulting in selective tissue-specific attenuation and nonhuman-range restriction of live attenuated vaccine viruses (Tsetsarkin et al., 2016a; Tsetsarkin et al., 2016b).

## Concluding Remarks

Currently, miRNA-based approaches employ four steps that address 20 aspects as listed in **Figure 1**. These exploratory studies are limited because of the bottleneck at the preapplication investigation step (**Figure 1**) and require further advances towards field or clinical applications. Twenty-four mosquito species have been analyzed for miRNA-related studies. The study materials have ranged widely, from entire mosquitoes to the cytoplasm or nucleus of mosquito cells, from eggs to adult mosquitoes, or from sugar-fed to pathogen-infected mosquitoes (**Tables 1** **–** **6**).

The expression of miRNAs is regulated by complex factors, including mosquito species, sex, developmental stage, tissue or organ, age, blood feeding status, pathogen infection status and pathogen type (**Tables 2** **–** **7**). Thus, miRNA expression levels detected in entire mosquitoes may lead to biased results, and for one arbovirus, some miRNAs may promote infection in mosquitoes, while for another arbovirus, miRNAs may inhibit infection (**Table 7**). Thus, during the exploration of miRNA-based approaches for the interruption of mosquito-borne disease transmission, an irrational approach is to commonly define a miRNA as solely inhibiting or promoting pathogen infection in mosquitoes, when the actual effects of a miRNA depend on those complex factors. Most importantly, the results presented here indicate that the selection of a candidate miRNA according to unique conditions or objectives during miRNA-based approach development is crucial. The current statuses of individual miRNAs presented in **Tables 1** **–** **7** and **S1** provide guidance for selection.

**Figure 2:**
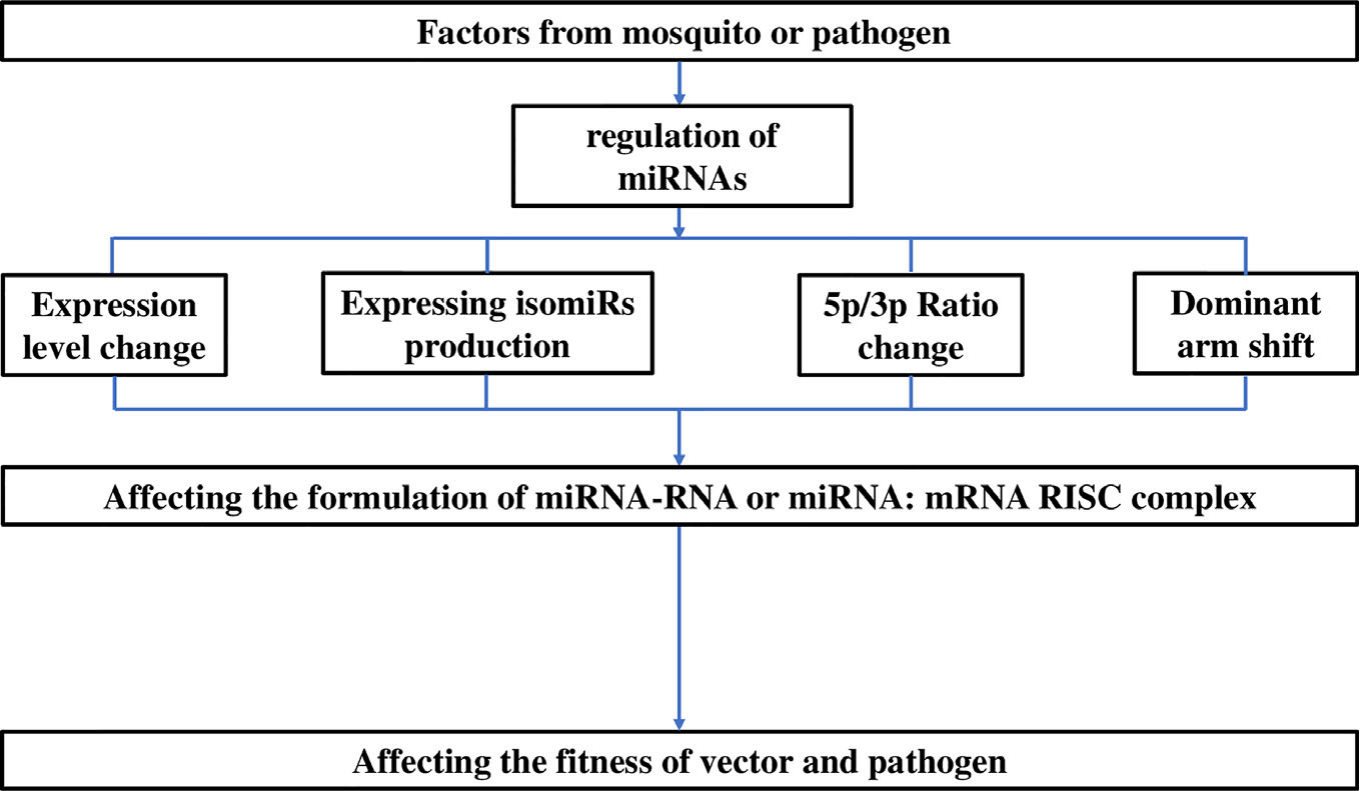
Responses of miRNAs to characteristics of mosquitoes or pathogens and subsequent miRNA-pathogen interactions in mosquitoes. The responses involve modifications in the expression level, isomiR production, or 5p/3p ratio or shift in dominant arm, and any of these alterations can affect the fitness between the vector and pathogen. During modification by miRNAs, the functional component is the formation of the miRNA-target RISC complex. For arboviruses, the complex can be composed of miRNAs and the virus genome (miR-RNA complex) in mosquitoes, directly regulating pathogen infection; however, for *Plasmodium* parasites, it is always composed of miRNAs and mosquito immunity-related mRNAs (miR-mRNA complex), indirectly regulating the infection.

As described above, the main variations in miRNAs attributed to the mosquito species or infecting pathogen include changes in the expression level, isomiR production, or 5p/3p ratio or even a shift in the dominant arm. In our opinion, these variations in miRNAs might collectively or individually affect the formulation of miRNA-target RISC complexes, and subsequently influence the fitness between the mosquito and pathogen (Winter et al., 2007). And for mosquito miRNA-arbovirus interactions, the targets of miRNAs can be RNA genomes of arboviruses, which are always mRNA obeying the canonical action mechanism. These viewpoints are presented in **Figure 2**.

**Figure 3:**
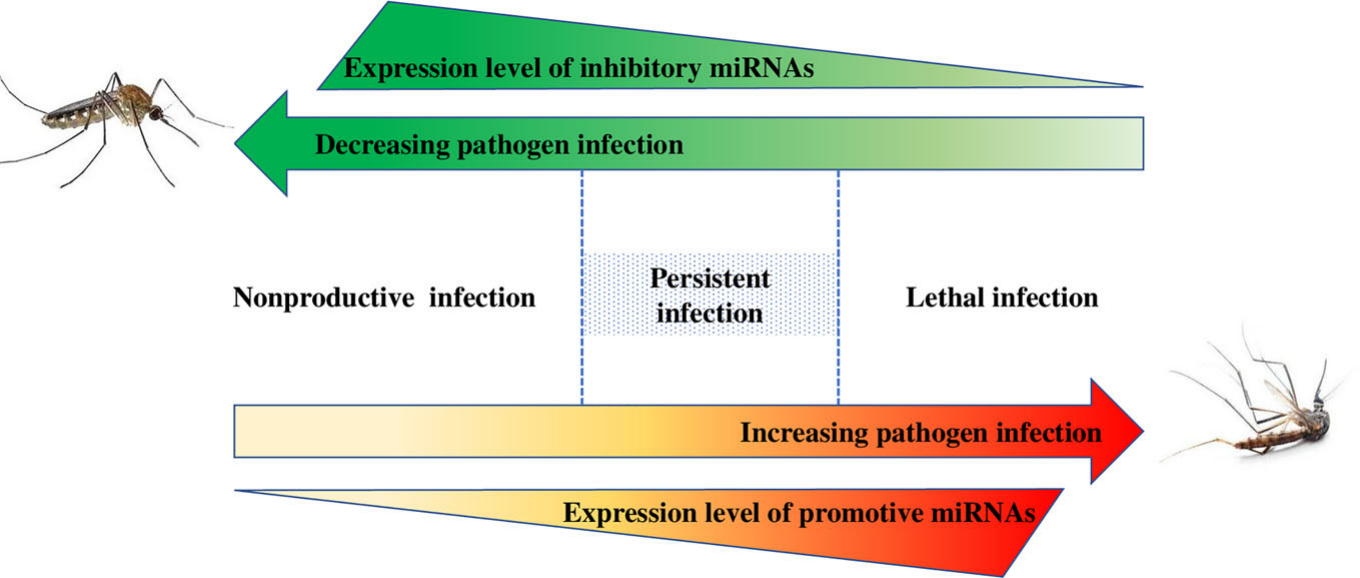
Causal relationship between mosquito miRNAs and pathogen infection. The manipulation by miRNAs is mediated *via* a network. The inhibitory miRNAs and promotive miRNAs always coexist in mosquitoes, inhibiting or promoting pathogen infection, respectively. These miRNAs can balance the miRNA-pathogen interaction, maintain persistent infection and preventing considerable harm to the mosquito.

Moreover, although the canonical action mechanism of miRNAs always results in repression, the mosquito miRNA-target interaction can lead to two possible forms of regulation, namely, repression or enhancement of pathogen infection in mosquitoes. Both upregulation and downregulation of miRNAs in response to pathogen infection widely coexist in the mosquito, subsequently promoting and inhibiting pathogen infection, respectively. In our opinion, these findings suggest that inhibitory and inducing miRNA expressions are essential to balance the miRNA-pathogen interaction, maintaining persistent infection and preventing considerable harm to the mosquito (**Figure 3**).

Currently, the antiviral effects of mosquito miRNAs on pathogens in combination with genetic engineering and molecular biology techniques may allow the use of these miRNA-based approaches as new tools to interrupt the transmission of mosquito-borne diseases. In this review, the significant progress achieved at the level of individual miRNAs facilitates the selection of an abundant, specific and effective mosquito miRNA (see **Tables 1** **–** **7** and **S1**) that can be referenced for further research with different and specific objectives to increase the pace of development of applications and overcome the bottleneck (Tsetsarkin et al., 2015). More importantly, mosquito miRNAs can directly bind to the arbovirus genome, modifying viral replication. However, regarding the *Plasmodium* parasite, mosquito miRNAs generally bind to mosquito immunity or development-related mRNAs, indirectly regulating *Plasmodium* infection. Hence, the strategies for miRNA-based approaches differ for arboviruses and protozoan parasites.

## Author Contributions

Conceptualization and formal analysis: T-LX. Data curation: T-LX, Y-WS, and X-YF.

Supervision: BZ and X-NZ. Writing-original draft: T-LX. Writing-review & editing: BZ and X-NZ. All authors contributed to the article and approved the submitted version.

## Funding

BZ received a grant from the National Science and Technology Major Program of China (No. 2018ZX10734-404). This project was financially supported by Ministry of Science and Technology of the People’s Republic of China. The funder had no role in study design, data collection and analysis, decision to publish, or preparation of the manuscript.

## Conflict of Interest

The authors declare that the research was conducted in the absence of any commercial or financial relationships that could be construed as a potential conflict of interest.
