# Supplementary material for: Development of miRNA-Based Approaches to Explore the Interruption of Mosquito-Borne Disease Transmission
Source: Front Cell Infect Microbiol. 2021 Jun 21;11:665444. doi: 10.3389/fcimb.2021.665444 (PMC8256169; doi:10.3389/fcimb.2021.665444)
Supplement: Supplementary file 1 [file Table_1.docx]

**Table S1. Predicted and validated of mosquito miRNA targets and functions**

| **miRNA names*** | **Predicted target** | **Validated target** | **Predicted function** | **Validated function** |
| --- | --- | --- | --- | --- |
| *miR-1-3p* | Capsid of DENV-1, NS5 and NS2A regions of DENV-4 (Yen et al., 2019), TDP-43 (Fu et al., 2017) | — | Increasing immunity (Batz et al., 2017) | — |
| miR-10-5p | — | — | Regulation of Hox genes (Dritsou et al., 2014) | — |
| miR-100-5p | Chemokine receptor [CXCR7](https://www.proteinatlas.org/ENSG00000144476-ACKR3/tissue) (Bruno et al., 2019) | — | Citrate cycle, dorso-ventral axis formation, valine, leucine and isoleucine degradation (Shrinet et al., 2014) | — |
| miR-1000-5p | Protein processing in endoplasmic reticulum, ribosome pathway (Shrinet et al. 2014) | — | — | — |
| **miR-11-3p** | NS4B of DENV-1 (Yen et al. 2019), 3’UTR of [PGRP-LC](https://www.ncbi.nlm.nih.gov/gene/?term=AGAP005203) (Dennison et al., 2015) | [AAEL013070](https://www.ncbi.nlm.nih.gov/gene/?term=AAEL013070) (Zhang et al., 2017), NS4B of DENV-1 (Yen et al. 2019) | Signal transduction mechanisms, intracellular trafficking, secretion, and vesicular transport, energy production and conversion (Zhang et al. 2017) | Removing protein phosphate groups in lysosomes during blood digestion (Zhang et al. 2017), |
| **miR-1174-3p** | — | SHMT (Liu et al., 2014) | — | Fluid excretion, blood intake and absorption, egg maturation (Liu et al. 2014) |
| miR-1175-3p | Exportin-5 (Jain et al., 2014) | — | Proteasome, RNA transport, protein processing in endoplasmic reticulum and N-glycan biosynthesis (Jain et al. 2014) | — |
| **miR-12-5p** | — | MCM6 and MCT1 (Osei-Amo et al., 2012) | Differentiation and maintenance of tissue identity (Winter et al., 2007) | Facilitating the *Wolbachia* infection (Osei-Amo et al. 2012, Maharaj et al., 2015), enhancing CHIKV infection (Maharaj et al. 2015) |
| ***miR-124-3p*** | Immune, pentose phosphate pathway, citrate cycle, oxidative phosphorylation, carbon metabolism, protein processing in endoplasmic reticulum (Jain et al. 2014), [PGRP-LD](https://www.ncbi.nlm.nih.gov/gene/?term=PGRP-LD) (Feng et al., 2018), 8 KEGG pathways (Meuti et al., 2018), NS4A of DENV-3 (Yen et al. 2019) | Dynamin 2 (Yang et al., 2016), [PGRP-LD](https://www.ncbi.nlm.nih.gov/gene/?term=PGRP-LD) (Feng et al. 2018) | Facilitating *Plasmodium* development and transmission to mammalian hosts (Jain et al. 2014), maintaining the diapause phenotype (Meuti et al. 2018) | Inhibiting JEV infection (Yang et al. 2016) |
| **miR-125-5p** | IRF4, PRDM1, BLIMP1/TNFAIP3, TNFα (Yen et al. 2019) | — | — | Enhancing CHIKV infection (Maharaj et al. 2015) |
| **miR-13-3p** | 8 KEGG pathways (Meuti et al. 2018) | CYP9J35 (Guo et al., 2017) | — | Regulating mosquito resistance to deltamethrin (Guo et al. 2017) |
| ***miR-137-3p*** | TGF-beta signaling pathway (Jain et al. 2014) | — | Immunity and protein processing in endoplasmic reticulum (Jain et al. 2014) | Inhibiting *Plasmodium* infection (Jain et al. 2014) |
| **miR-13664** | — | CpCYP314A1 (Sun et al., 2019) | — | Regulating deltamethrin resistance (Sun et al. 2019) |
| **miR-14-3p** | 8 KEGG pathways (Meuti et al. 2018) | 3’UTR-binding sites in the GCE mRNA (Qu et al., 2017) | Blood meal activated egg development of mosquitoes (Bryant et al., 2010), iapause entry and/or the switch from a developmental pathway (Meuti et al. 2018) | *P. falciparum* and gut microbiota agonist (Dong et al., 2020) |
| **miR-14-5p** | — | — | — | lipid accumulation (Batz et al. 2017) |
| **miR-1767** | — | — | — | Enhancing DENV-2 replication (Su et al., 2019) |
| ***miR-184-3p*** | FOXL (Nouzova et al., 2018) | [AAEL002512](https://www.ncbi.nlm.nih.gov/gene/?term=AAEL002512), [AAEL005741](https://www.ncbi.nlm.nih.gov/gene/?term=AAEL005741) (Zhang et al. 2017) | Egg development (Nouzova et al. 2018) | homeostasis of FOXL, egg development (Zhang et al. 2017), inhibiting CHIKV infection (Maharaj et al. 2015) |

| **Table S1. *Continued*** | | | | |
| --- | --- | --- | --- | --- |
| **miRNA names*** | **Predicted target** | **Validated target** | **Predicted function** | **Validated function** |
| miR-1890-3p | NS5 region of DENV-2 (Yen et al. 2019), 3’ UTR of JHA15 (Lucas et al., 2015) | — | — | — |
| **miR-1891-5p** | — | Exportin-5 (Jain et al. 2014) | — | Ovulation development or blood digestion (Puthiyakunnon et al., 2013) |
| *miR-210-3p* | AKH2 (Nouzova et al. 2018) | — | Protein processing in endoplasmic reticulum (Jain et al. 2014) | — |
| miR-219-5p | NS5 and NS2A regions of DENV-4 (Yen et al. 2019) | — | — | — |
| **miR-252-5p** | — | DENV E protein gene (Yan et al., 2014) |  | Inhibiting DENV replication (Yan et al. 2014) |
| ***miR-275-3p*** | 3'UTR of PGRP-LB and 3'-UTR of anti-*Plasmodium* genes, such as [PGRP-LC](https://www.ncbi.nlm.nih.gov/gene/?term=AGAP005203), [PGRP-LD](https://www.ncbi.nlm.nih.gov/gene/?term=PGRP-LD), [Rel2](https://www.ncbi.nlm.nih.gov/gene?term=(rel2%5bgene%5d)%20AND%20(Anopheles%20gambiae%20str.%20PEST%5borgn%5d)%20AND%20alive%5bprop%5d%20NOT%20newentry%5bgene%5d&sort=weight), [Caspar](https://www.ncbi.nlm.nih.gov/gene/36769), [IMD](https://www.ncbi.nlm.nih.gov/gene?term=(imd%5bgene%5d)%20AND%20(Anopheles%20gambiae%20str.%20PEST%5borgn%5d)%20AND%20alive%5bprop%5d%20NOT%20newentry%5bgene%5d&sort=weight) and LRRD7/APL2 (Feng et al. 2018), 8 KEGG pathways (Meuti et al. 2018) | Sarco/endoplasmic reticulum Ca2+ adenosine triphosphatase (Zhao et al., 2017) | — | Maintenance of multiple gut functions (Zhao et al. 2017), blood digestion, fluid excretion and egg development(Bryant et al. 2010, Jain et al. 2014) |
| miR-275-5p | Information storage and processing, cellular processing and signaling, amino acid and nucleotide transport and metabolism (Zhang et al. 2017) | — |  | — |
| **miR-276-5p** | — | Branched-chain amino acid transferase (Lampe et al., 2019) | — | Enhancing DENV 2 infection (Su et al. 2019), prolonging AA catabolism, and then inhibiting sporozoite development (Lampe et al. 2019) |
| **miR-277-3p** | Genes encoding Netrin-B, starry night, visceral mesodermal armadillo-repeats (Thirugnanasambantham et al., 2013), 8 KEGG pathways (Meuti et al. 2018) | Insulin-like peptides 7 and 8 (Ling et al., 2017) | Axon guidance and rhabdomere development, cell proliferation, lipid metabolism, signal transduction, and response to light stimulus (Thirugnanasambantham et al. 2013, Zhang et al. 2017), energy production and conversion(Zhang et al. 2017) | Regulating lipid metabolism and subsequent lipid storage and ovary development (Ling et al. 2017) |
| **miR-278-3p** | AaArgM3 (Zhang et al., 2014), JHAMT 3’UTR or Met orthologues (Qu et al. 2017), [PGRP-LD](https://www.ncbi.nlm.nih.gov/gene/?term=PGRP-LD) (Feng et al. 2018), CYP6AG11 (Lei et al., 2015) | CYP6AG11 (Lei et al. 2015) | — | Regulating pyrethroid resistance (Guo et al. 2017) |
| ***miR-279-3p*** | CHIKV (Yen et al. 2019) | [AAEL010015](https://www.ncbi.nlm.nih.gov/gene/?term=AAEL010015) (Zhang et al. 2017) | — | — |
| **miR-281-3p** | NS5 and NS2A regions of DENV-4 (Yen et al. 2019) | 5'-UTR of DENV-2 (Zhou et al., 2014) | — | Enhancing DENV-2 replication (Zhou et al. 2014) |
| miR-282-5p | [PGRP-LD](https://www.ncbi.nlm.nih.gov/gene/?term=PGRP-LD) (Feng et al. 2018), minus strand of CHIKV RNA genome (Yen et al. 2019), the NS3 protein coding regions of DENV-1 (Yen et al. 2019) | — | — | — |
| **miR-285-3p** | AKH2 (Nouzova et al. 2018), E1 of the CHIKV (Yen et al. 2019), protein processing in endoplasmic reticulum and glutathione metabolic pathway (Jain et al. 2014) | CYP6N23 (Tian et al., 2016) | — | Increasing *Cx. pipiens* pallens mosquito resistance to deltamethrin (Tian et al. 2016) |
| miR-286-3p | 5' or 3' UTR of ZIKV (Saldana et al., 2017, Yen et al. 2019) | — | — | — |
| miR-286b-3p | 5' or 3' UTR of ZIKV, NS5 region of ZIKA (Yen et al. 2019) | — | — | — |

| **Table S1. *Continued*** | | | | |
| --- | --- | --- | --- | --- |
| **miRNA names*** | **Predicted target** | **Validated target** | **Predicted function** | **Validated function** |
| **miR-2940-5p** | GS1 ([AAEL001887](https://www.ncbi.nlm.nih.gov/gene/?term=AAEL001887)) (Zhang et al. 2017) | [AaDnmt2](https://www.ncbi.nlm.nih.gov/gene/?term=XM_001657505) (Zhang et al., 2013), metalloprotease m41 FtsH gene (Slonchak et al., 2014), AaArgM3 (Zhang et al. 2014) | Ammonia detoxification (Zhang et al. 2017) | Facilitating *Wolbachia* infection (Hussain et al., 2011, Zhang et al. 2014) and subsequent inhibition of DENV replication (Zhang et al. 2013), restricting WNV replication (Slonchak et al. 2014) |
| miR-2941-3p | [LRR gene](https://www.ncbi.nlm.nih.gov/gene?term=(lrr%5bgene%5d)%20AND%20(Drosophila%20melanogaster%5borgn%5d)%20AND%20alive%5bprop%5d%20NOT%20newentry%5bgene%5d&sort=weight), IRGs encoding recognition, modulation, signal transduction and effector molecules (Liu et al., 2016) | — | — | — |
| **miR-2942-3p** | — | — | — | Eclosion of larva (Puthiyakunnon et al. 2013, Feng et al. 2018) |
| miR-2943-5p | LYS gene, IRGs encoding recognition, modulation, signal transduction and effector molecules (Liu et al. 2016) | — | — | — |
| **miR-2944a-5p** | SHP2 phosphatase, signaling, metabolic pathways (Shrinet et al. 2014) | vps-13 and CHIKV (Dubey et al., 2019) | — | Repressing CHIKV replication (Dubey et al. 2019) |
| **miR-2c-3p** | 3’-UTR of the *P. falciparum* antagonists IMD (Dennison et al. 2015) | CYP9J35 (Guo et al. 2017) | Anti-*Plasmodium* (Dennison et al. 2015) | Increasing resistance to deltamethrin (Guo et al. 2017) |
| **miR-305-5p** | TOR signaling pathway [44], pentose phosphate pathway, citrate cycle and oxidative phosphorylation (Jain et al. 2014), ZIKA viral genome (Saldana et al. 2017), AKH1 and corazonin (Nouzova et al. 2018), 3'UTR of PGRP-LB (Feng et al. 2018) | — | Activating yolk protein precursors for egg development [44] | *P. falciparum* and gut microbiota agonist (Dennison et al. 2015, Dong et al. 2020) |
| miR-305-3p | Citrate cycle, dorso-ventral axis formation, valine, leucine and isoleucine degradation (Shrinet et al. 2014), 8 KEGG pathways (Meuti et al. 2018) | — | — | — |
| **miR-309a-3p** | — | SIX4 (Zhang et al., 2016, Fu et al. 2017, Fu et al., 2020), | — | Regulating mosquito reproduction (Zhang et al. 2016, Fu et al. 2017, Fu et al. 2020) |
| miR-31-5p | Endocytosis and fructose mannose metabolism pathway (Guo et al. 2017), HMGR, AKH1 (Nouzova et al. 2018) | — | Functioning as a tumour suppressor (Liu et al., 2014) | — |
| **miR-315-5p** | 3’UTR of JHA15 (Yen et al. 2019) | GFP or AaArgM3 (Zhang et al. 2014) | — | — |
| miR-316-5p | NS5 region of DENV-1, capsid region of DENV 1-3 (Yen et al. 2019), posttranslational modification, protein turnover, chaperones, lipid, amino acid, nucleotide and coenzyme transport and metabolism (Zhang et al. 2017) | — | — | — |
| **miR-317-3p** | Posttranslational modification, protein turnover, chaperones, lipid, amino acid, nucleotide and coenzyme transport and metabolism (Zhang et al. 2017) | — | — | Regulating stability of AGO1 (Hussain et al., 2013) |

| **Table S1. *Continued*** | | | | |
| --- | --- | --- | --- | --- |
| **miRNA names*** | **Predicted target** | **Validated target** | **Predicted function** | **Validated function** |
| **miR-34-5p** | 3’-UTR of REL1, Caspar, Cecropin3 (Dennison et al. 2015), Toll-like receptor signaling pathway, defense response, immune response, innate immune response, DEFD (AAEL003857), PGRP-LE (AAEL013112), Toll-like receptor signaling pathway (Liu et al., 2015), PP-303 MevD, ALDH, AKH2, CCAP (Nouzova et al. 2018) | Activation of type I interferon signaling (Smith et al., 2017) | Regulating anti-pathogen and immune responses in DENV-2 infected *Ae. albopictus* (Liu et al. 2015) | Role in vector competence (Winter et al. 2007), inhibits multiple flaviviruses (Smith et al. 2017) |
| miR-34-3p | 5' or 3' UTR ZIKV (Yen et al. 2019) | — | — | — |
| **miR-375-3p** | Toll pathway (Saldana et al. 2017) | Cactus, kinesin, prohibitin, DEAD box ATP-depen-dent RNA helicase, REL1, hypothetical protein (Hussain et al. 2013) | — | Enhancing DENV-2 infection (Hussain et al. 2013), repressing CHIKV replication (Maharaj et al. 2015). |
| **miR-4448** | — | — | — | Inhibiting DENV-2 infection (Su et al. 2019) |
| **miR-4728-5p** | — | — | — | Enhancing DENV infections (Su et al., 2017) |
| miR-7-5p | EG FR, IGF1R, NF-kB/RelA, RNF183 (Bruno et al. 2019) | — | — | — |
| **miR-71-3p** | — | CYP325BG3 (Hong et al., 2014, Guo et al. 2017) | — | Regulating resistance to deltamethrin (Guo et al. 2017) |
| miR-79-5p | Capsid region of DENV-3 (Yen et al. 2019) | — | — | — |
| **miR-8-3p** | Met-1 3’UTR (Qu et al. 2017), posttranslational modification, protein turnover, chaperones, lipid transport and metabolism (Zhang et al. 2017) | SWIM (Lucas et al., 2015), CLIP-Seq (Zhang et al. 2017) | — | Egg development and deposition (Lucas et al. 2015) |
| miR-8-5p | AKH1 (Nouzova et al. 2018) | — | — | — |
| miR-87-3p | Toll-like receptor signaling pathway, defense response, immune response, innate immune response (Liu et al. 2015) | — | Anti-pathogen and immune responses (Liu et al. 2015) | — |
| **miR-927-5p** | SNARE interactions in vesicular transport (Shrinet et al. 2014), SUMO, eIF-2B, NKIRAS, EXOC-2, APM1, FLN (Avila-Bonilla et al., 2020) | FLN (Avila-Bonilla et al. 2020) | Post translational modifications, translation factors, innate immune system, exocytosis, endocytosis, cytoskeleton (Avila-Bonilla et al. 2020) | Reguating antimicrobial peptides, promoting DENV infection (Avila-Bonilla et al. 2020) |
| **miR-92a-3p** | NS5 region of DENV-1 (Yen et al. 2019) | CpCPR4 (Ma et al., 2017) | — | Regulating deltamethrin resistance (Ma et al. 2017) |
| **miR-932-5p** | Pentose phosphate pathway and citrate cycle (Jain et al. 2014) | CpCPR5 (Liu et al., 2016) | — | Regulating deltamethrin resistance (Liu et al. 2016) |
| **miR-981-3p** | RNA transport (Jain et al. 2014) | Importin β-4 (Hussain et al., 2013) | — | Mediating AGO1 intracellular translocation to nucleus (Hussain et al. 2013) |
| miR-989-3p | Carbon metabolism and protein processing in endoplasmic reticulum (Jain et al. 2014), MYD88, DEF, SRPN, GALE (galectin) and FREP (Liu et al. 2016), AGAP007839-RA, AGAP0004451-RA, AGAP008345-RA (Guo et al. 2017), Capsid coding regions of CHIKV (Yen et al. 2019) | — | Regulation of DENV infection. (Liu et al. 2016) | — |

**Table S1. *Continued***

| **miRNA names*** | **Predicted target** | **Validated target** | - **Predicted function** | **Validated function** |
| --- | --- | --- | --- | --- |
| miR-996-3p | CHIKV (Yen et al. 2019) | — |  | — |
| miR-998-3p | AKH2 (Nouzova et al. 2018) | — | — | — |
| miR-9c-5p | DENV-2 (Yen et al. 2019) | — | — | — |
| miR-iab-4-5p | 3’ UTR of CHIKV (Yen et al. 2019) | — | — | — |
| bantam-3p | AcCoAS (AAEL015010, AAEL000321), HDAC RPD3 orthologue (AAEL004586) (Zhang et al. 2017), PP-MevD (Nouzova et al. 2018) | — | Involving insulin and ecdysone signaling in *An. gambiae* females (Biryukova et al., 2014) | — |
| ***let-7-5p*** | CCAP (Nouzova et al. 2018) | Kr-h1(Fu et al. 2020) | Hormonal signals (Winter et al. 2007), CC neuropeptides (Nouzova et al. 2018) | Regulating egg development (Fu et al. 2020) |

Notes: *: The targets or functions of miRNAs in **bold** font have been verified experimentally, and the miRNA names presented in *italic* have entered into the step of preapplication investigation; TDP-43: TAR DNA-binding protein-43; 8 KEGG pathways: Mucin type O-Glycan biosynthesis, Valine, leucine and isoleucine degradation, Fatty acid elongation, Fatty acid degradation, Propanoate metabolism, MAPK signaling pathway, Hippo signaling pathway, and Valine, leucine and isoleucine biosynthesis; MCT1: monocarboxylate transporter; MCM6: DNA replication licensing; CYP9J35: cytochrome P450 9J35; IMD: immune deficiency pathway; PP-MevD: phosphomevalonate decarboxylase; HDAC: a histone deacetylase; AcCoAS: acetyl-CoA synthetase; FREP: fibrinogen and fibronectin; DEFD: antimicrobial peptide defensin D; PGRP-LE: peptidoglycan recognition protein LE; HMGR: 3-hydroxy-3-methylglutaryl-coenzyme A reductase; swim: wingless interacting molecule; UTR: untranslated region; SHMT: hydroxymethyltransferase; CpCPR4: the cuticular protein gene; SUMO: posttranslational modifications; eIF-2B: translation factor; NKIRAS: gene in the innate immune system; EXOC-2: exocytosis; APM1: endocytosis; FLN: the cytoskeleton; Kr-h1: Krüppel-homolog 1; AA: amino acid; SIX4: homeobox 4 protein; AaArgM3: protein arginine methyltransferase 3; IRGs: immune related genes; JHA15: juvenile hormone-regulated serine protease JHA15.

**References**

Avila-Bonilla, R. G., M. Yocupicio-Monroy, L. A. Marchat, D. G. Perez-Ishiwara, D. A. Cerecedo-Mercado, R. M. Del Angel, et al. (2020). miR-927 has pro-viral effects during acute and persistent infection with dengue virus type 2 in C6/36 mosquito cells. *J Gen Virol* 101, 825-839. doi: 10.1099/jgv.0.001441.

Batz, Z. A., A. C. Goff and P. A. Armbruster. (2017). MicroRNAs are differentially abundant during *Aedes albopictus* diapause maintenance but not diapause induction. *Insect Mol Biol* 26, 721-733. doi: 10.1111/imb.12332.

Biryukova, I., T. Ye and E. Levashina. (2014). Transcriptome-wide analysis of microRNA expression in the malaria mosquito *Anopheles gambiae*. *BMC Genomics* 15, 557. doi: 10.1186/1471-2164-15-557.

Bruno, A., C. Alessio, F. Carmine, S. Francesco, B. Vladimir, D. L. Marco, et al. (2019). MicroRNAs from saliva of *anopheline* mosquitoes mimic human endogenous miRNAs. *Scientific reports* 9, 2955. doi: 10.1038/s41598-019-39880-1.

Bryant, B., W. Macdonald and A. S. Raikhel. (2010). microRNA miR-275 is indispensable for blood digestion and egg development in the mosquito *Aedes aegypti*. *Proc Natl Acad Sci U S A* 107, 22391-22398. doi: 10.1073/pnas.1016230107.

Dennison, N. J., O. J. BenMarzouk-Hidalgo and G. Dimopoulos. (2015). MicroRNA-regulation of *Anopheles gambiae* immunity to *Plasmodium falciparum* infection and midgut microbiota. *Dev Comp Immunol* 49, 170-178. doi: 10.1016/j.dci.2014.10.016.

Dong, S., X. Fu, Y. Dong, M. L. Simoes, J. Zhu and G. Dimopoulos. (2020). Broad spectrum immunomodulatory effects of *Anopheles gambiae* microRNAs and their use for transgenic suppression of *Plasmodium*. *PLoS Pathog* 16, e1008453. doi: 10.1371/journal.ppat.1008453.

Dritsou, V., E. Deligianni, E. Dialynas, J. Allen, N. Poulakakis, C. Louis, et al. (2014). Non-coding RNA gene families in the genomes of *anopheline* mosquitoes. *BMC Genomics* 15, 1038. doi: 10.1186/1471-2164-15-1038.

Dubey, S. K., J. Shrinet and S. Sunil. (2019). *Aedes aegypti* microRNA, miR-2944b-5p interacts with 3'UTR of chikungunya virus and cellular target vps-13 to regulate viral replication. *PLoS Negl Trop Dis* 13, e0007429. doi: 10.1371/journal.pntd.0007429.

Feng, X., J. Wu, S. Zhou, J. Wang and W. Hu. (2018). Characterization and potential role of microRNA in the Chinese dominant malaria mosquito *Anopheles sinensis* (Diptera: Culicidae) throughout four different life stages. *Cell Biosci* 8, 29. doi: 10.1186/s13578-018-0227-1.

Fu, X., G. Dimopoulos and J. Zhu. (2017). Association of microRNAs with Argonaute proteins in the malaria mosquito *Anopheles gambiae* after blood ingestion. *Sci Rep* 7, 6493. doi: 10.1038/s41598-017-07013-1.

Fu, X., P. Liu, G. Dimopoulos and J. Zhu. (2020). Dynamic miRNA-mRNA interactions coordinate gene expression in adult *Anopheles gambiae*. *PLoS Genet* 16, e1008765. doi: 10.1371/journal.pgen.1008765.

Guo, Q., Y. Huang, F. Zou, B. Liu, M. Tian, W. Ye, et al. (2017). The role of miR-2~13~71 cluster in resistance to deltamethrin in *Culex pipiens* pallens. *Insect Biochem Mol Biol* 84, 15-22. doi: 10.1016/j.ibmb.2017.03.006.

Hong, S., Q. Guo, W. Wang, S. Hu, F. Fang, Y. Lv, et al. (2014). Identification of differentially expressed microRNAs in *Culex pipiens* and their potential roles in pyrethroid resistance. *Insect Biochem Mol Biol* 55, 39-50. doi: 10.1016/j.ibmb.2014.10.007.

Hussain, M., F. D. Frentiu, L. A. Moreira, S. L. O'Neill and S. Asgari. (2011). *Wolbachia* uses host microRNAs to manipulate host gene expression and facilitate colonization of the dengue vector *Aedes aegypti*. *Proc Natl Acad Sci U S A* 108, 9250-9255. doi: 10.1073/pnas.1105469108.

Hussain, M., S. L. O'Neill and S. Asgari. (2013). *Wolbachia* interferes with the intracellular distribution of Argonaute 1 in the dengue vector *Aedes aegypti* by manipulating the host microRNAs. *RNA Biol* 10, 1868-1875. doi: 10.4161/rna.27392.

Hussain, M., T. Walker, S. L. O'Neill and S. Asgari. (2013). Blood meal induced microRNA regulates development and immune associated genes in the Dengue mosquito vector, *Aedes aegypti*. *Insect Biochem Mol Biol* 43, 146-152. doi: 10.1016/j.ibmb.2012.11.005.

Jain, S., V. Rana, J. Shrinet, A. Sharma, A. Tridibes, S. Sunil, et al. (2014). Blood feeding and *Plasmodium* infection alters the miRNome of *Anopheles stephensi*. *PLoS One* 9, e98402. doi: 10.1371/journal.pone.0098402.

Lampe, L., M. Jentzsch, S. Kierszniowska and E. A. Levashina. (2019). Metabolic balancing by miR-276 shapes the mosquito reproductive cycle and *Plasmodium falciparum* development. *Nat Commun* 10, 5634. doi: 10.1038/s41467-019-13627-y.

Lei, Z., Y. Lv, W. Wang, Q. Guo, F. Zou, S. Hu, et al. (2015). MiR-278-3p regulates pyrethroid resistance in *Culex pipiens* pallens. *Parasitol Res* 114, 699-706. doi: 10.1007/s00436-014-4236-7.

Ling, L., V. A. Kokoza, C. Zhang, E. Aksoy and A. S. Raikhel. (2017). MicroRNA-277 targets insulin-like peptides 7 and 8 to control lipid metabolism and reproduction in *Aedes aegypti* mosquitoes. *Proc Natl Acad Sci U S A* 114, E8017-E8024. doi: 10.1073/pnas.1710970114.

Liu, B., M. Tian, Q. Guo, L. Ma, D. Zhou, B. Shen, et al. (2016). MiR-932 Regulates Pyrethroid Resistance in *Culex pipiens* pallens (Diptera: Culicidae). *J Med Entomol* 53, 1205-1210. doi: 10.1093/jme/tjw083.

Liu, S., K. J. Lucas, S. Roy, J. Ha and A. S. Raikhel. (2014). Mosquito-specific microRNA-1174 targets serine hydroxymethyltransferase to control key functions in the gut. *Proc Natl Acad Sci U S A* 111, 14460-14465. doi: 10.1073/pnas.1416278111.

Liu, W., H. Huang, C. Xing, C. Li, F. Tan and S. Liang. (2014). Identification and characterization of the expression profile of microRNAs in *Anopheles anthropophagus*. *Parasit Vectors* 7, 159. doi: 10.1186/1756-3305-7-159.

Liu, Y., Y. Zhou, J. Wu, P. Zheng, Y. Li, X. Zheng, et al. (2015). The expression profile of *Aedes albopictus* miRNAs is altered by dengue virus serotype-2 infection. *Cell Biosci* 5, 16. doi: 10.1186/s13578-015-0009-y.

Liu, Y. X., F. X. Li, Z. Z. Liu, Z. R. Jia, Y. H. Zhou, H. Zhang, et al. (2016). Integrated analysis of miRNAs and transcriptomes in *Aedes albopictus* midgut reveals the differential expression profiles of immune-related genes during dengue virus serotype-2 infection. *Insect Sci* 23, 377-385. doi: 10.1111/1744-7917.12339.

Lucas, K. J., S. Roy, J. Ha, A. L. Gervaise, V. A. Kokoza and A. S. Raikhel. (2015). MicroRNA-8 targets the Wingless signaling pathway in the female mosquito fat body to regulate reproductive processes. *Proc Natl Acad Sci U S A* 112, 1440-1445. doi: 10.1073/pnas.1424408112.

Lucas, K. J., B. Zhao, S. Roy, A. L. Gervaise and A. S. Raikhel. (2015). Mosquito-specific microRNA-1890 targets the juvenile hormone-regulated serine protease JHA15 in the female mosquito gut. *RNA Biol* 12, 1383-1390. doi: 10.1080/15476286.2015.1101525.

Ma, K., X. Li, H. Hu, D. Zhou, Y. Sun, L. Ma, et al. (2017). Pyrethroid-resistance is modulated by miR-92a by targeting CpCPR4 in *Culex pipiens* pallens. *Comp Biochem Physiol B Biochem Mol Biol* 203, 20-24. doi: 10.1016/j.cbpb.2016.09.002.

Maharaj, P. D., S. G. Widen, J. Huang, T. G. Wood and S. Thangamani. (2015). Discovery of mosquito saliva microRNAs during CHIKV infection. *PLoS Negl Trop Dis* 9, e0003386. doi: 10.1371/journal.pntd.0003386.

Meuti, M. E., R. Bautista-Jimenez and J. A. Reynolds. (2018). Evidence that microRNAs are part of the molecular toolkit regulating adult reproductive diapause in the mosquito, Culex pipiens. *PLoS One* 13, e0203015. doi: 10.1371/journal.pone.0203015.

Nouzova, M., K. Etebari, F. G. Noriega and S. Asgari. (2018). A comparative analysis of corpora allata-corpora cardiaca microRNA repertoires revealed significant changes during mosquito metamorphosis. *Insect Biochem Mol Biol* 96, 10-18. doi: 10.1016/j.ibmb.2018.03.007.

Osei-Amo, S., M. Hussain, S. L. O'Neill and S. Asgari. (2012). Wolbachia-induced aae-miR-12 miRNA negatively regulates the expression of MCT1 and MCM6 genes in *Wolbachia*-infected mosquito cell line. *PLoS One* 7, e50049. doi: 10.1371/journal.pone.0050049.

Puthiyakunnon, S., Y. Yao, Y. Li, J. Gu, H. Peng and X. Chen. (2013). Functional characterization of three MicroRNAs of the Asian tiger mosquito, *Aedes albopictus*. *Parasit Vectors* 6, 230. doi: 10.1186/1756-3305-6-230.

Qu, Z., W. G. Bendena, W. Nong, K. W. Siggens, F. G. Noriega, Z. P. Kai, et al. (2017). MicroRNAs regulate the sesquiterpenoid hormonal pathway in *Drosophila* and other arthropods. *Proc Biol Sci* 284, 1869. doi: 10.1098/rspb.2017.1827.

Saldana, M. A., K. Etebari, C. E. Hart, S. G. Widen, T. G. Wood, S. Thangamani, et al. (2017). Zika virus alters the microRNA expression profile and elicits an RNAi response in *Aedes aegypti* mosquitoes. *PLoS Negl Trop Dis* 11, e0005760. doi: 10.1371/journal.pntd.0005760.

Shrinet, J., S. Jain, J. Jain, R. K. Bhatnagar and S. Sunil. (2014). Next generation sequencing reveals regulation of distinct *Aedes* microRNAs during chikungunya virus development. *PLoS Negl Trop Dis* 8, e2616. doi: 10.1371/journal.pntd.0002616.

Slonchak, A., M. Hussain, S. Torres, S. Asgari and A. A. Khromykh. (2014). Expression of mosquito microRNA Aae-miR-2940-5p is downregulated in response to West Nile virus infection to restrict viral replication. *J Virol* 88, 8457-8467. doi: 10.1128/JVI.00317-14.

Smith, J. L., S. Jeng, S. K. McWeeney and A. J. Hirsch. (2017). A MicroRNA Screen Identifies the Wnt Signaling Pathway as a Regulator of the Interferon Response during Flavivirus Infection. *J Virol* 91, e02388-02316. doi: 10.1128/JVI.02388-16.

Su, J., C. Li, Y. Zhang, T. Yan, X. Zhu, M. Zhao, et al. (2017). Identification of microRNAs expressed in the midgut of *Aedes albopictus* during dengue infection. *Parasit Vectors* 10, 63. doi: 10.1186/s13071-017-1966-2.

Su, J., G. Wang, C. Li, D. Xing, T. Yan, X. Zhu, et al. (2019). Screening for differentially expressed miRNAs in *Aedes albopictus* (Diptera: Culicidae) exposed to DENV-2 and their effect on replication of DENV-2 in C6/36 cells. *Parasit Vectors* 12, 44. doi: 10.1186/s13071-018-3261-2.

Sun, X. H., N. Xu, Y. Xu, D. Zhou, Y. Sun, W. J. Wang, et al. (2019). A novel miRNA, miR-13664, targets CpCYP314A1 to regulate deltamethrin resistance in *Culex pipiens* pallens. *Paras* 146, 197-205. doi: 10.1017/S0031182018001002.

Thirugnanasambantham, K., V. I. Hairul-Islam, S. Saravanan, S. Subasri and A. Subastri. (2013). Computational approach for identification of *Anopheles gambiae* miRNA involved in modulation of host immune response. *Appl Biochem Biotechnol* 170, 281-291. doi: 10.1007/s12010-013-0183-5.

Tian, M., B. Liu, H. Hu, X. Li, Q. Guo, F. Zou, et al. (2016). MiR-285 targets P450 (CYP6N23) to regulate pyrethroid resistance in Culex pipiens pallens. *Parasitol Res* 115, 4511-4517. doi: 10.1007/s00436-016-5238-4.

Winter, F., S. Edaye, A. Huttenhofer and C. Brunel. (2007). *Anopheles gambiae* miRNAs as actors of defence reaction against *Plasmodium* invasion. *Nucleic Acids Res* 35, 6953-6962. doi: 10.1093/nar/gkm686.

Yan, H., Y. Zhou, Y. Liu, Y. Deng and X. Chen. (2014). miR-252 of the Asian tiger mosquito *Aedes albopictus* regulates dengue virus replication by suppressing the expression of the dengue virus envelope protein. *J Med Virol* 86, 1428-1436. doi: 10.1002/jmv.23815.

Yang, S., Y. Pei, X. Li, S. Zhao, M. Zhu and A. Zhao. (2016). miR-124 attenuates Japanese encephalitis virus replication by targeting DNM2. *Virol J* 13, 105. doi: 10.1186/s12985-016-0562-y.

Yen, P. S., C. H. Chen, V. Sreenu, A. Kohl and A. B. Failloux. (2019). Assessing the Potential Interactions between Cellular miRNA and Arboviral Genomic RNA in the Yellow Fever Mosquito, *Aedes aegypti*. *Viruses* 11, 540. doi: 10.3390/v11060540.

Zhang, G., M. Hussain and S. Asgari. (2014). Regulation of arginine methyltransferase 3 by a *Wolbachia*-induced microRNA in *Aedes aegypti* and its effect on *Wolbachia* and dengue virus replication. *Insect Biochem Mol Biol* 53, 81-88. doi: 10.1016/j.ibmb.2014.08.003.

Zhang, G., M. Hussain, S. L. O'Neill and S. Asgari. (2013). *Wolbachia* uses a host microRNA to regulate transcripts of a methyltransferase, contributing to dengue virus inhibition in *Aedes aegypti*. *Proc Natl Acad Sci U S A* 110, 10276-10281. doi: 10.1073/pnas.1303603110.

Zhang, X., E. Aksoy, T. Girke, A. S. Raikhel and F. V. Karginov. (2017). Transcriptome-wide microRNA and target dynamics in the fat body during the gonadotrophic cycle of *Aedes aegypti*. *Proc Natl Acad Sci U S A* 114, E1895-E1903. doi: 10.1073/pnas.1701474114.

Zhang, Y., B. Zhao, S. Roy, T. T. Saha, V. A. Kokoza, M. Li, et al. (2016). microRNA-309 targets the Homeobox gene SIX4 and controls ovarian development in the mosquito *Aedes aegypti*. *Proc Natl Acad Sci U S A* 113, E4828-4836. doi: 10.1073/pnas.1609792113.

Zhao, B., K. J. Lucas, T. T. Saha, J. Ha, L. Ling, V. A. Kokoza, et al. (2017). MicroRNA-275 targets sarco/endoplasmic reticulum Ca2^+^ adenosine triphosphatase (SERCA) to control key functions in the mosquito gut. *PLoS Genet* 13, e1006943. doi: 10.1371/journal.pgen.1006943.

Zhou, Y., Y. Liu, H. Yan, Y. Li, H. Zhang, J. Xu, et al. (2014). miR-281, an abundant midgut-specific miRNA of the vector mosquito *Aedes albopictus* enhances dengue virus replication. *Parasit Vectors* 7, 488. doi: 10.1186/s13071-014-0488-4.
